# SP1‐activated USP27X‐AS1 promotes hepatocellular carcinoma progression via USP7‐mediated AKT stabilisation

**DOI:** 10.1002/ctm2.1563

**Published:** 2024-01-27

**Authors:** Chen Su, Haoquan Zhang, Jie Mo, Zhibin Liao, Bixiang Zhang, Peng Zhu

**Affiliations:** ^1^ Hepatic Surgery Center Tongji Hospital Tongji Medical College Huazhong University of Science and Technology Wuhan Hubei People's Republic of China; ^2^ Hubei Key Laboratory of Hepato‐Pancreato‐Biliary Diseases Wuhan Hubei People's Republic of China; ^3^ Key Laboratory of Organ Transplantation Ministry of Education Wuhan Hubei People's Republic of China; ^4^ Key Laboratory of Organ Transplantation National Health Commission Wuhan Hubei People's Republic of China; ^5^ Key Laboratory of Organ Transplantation Chinese Academy of Medical Sciences Wuhan Hubei People's Republic of China

**Keywords:** AKT, hepatocellular carcinoma, ubiquitination, USP27X‐AS1, USP7

## Abstract

**Background:**

Hepatocellular carcinoma (HCC) continues to pose a significant threat to patient survival. Emerging evidence underscores the pivotal involvement of long non‐coding RNAs (lncRNAs) in the cancer process. Nevertheless, our understanding of the roles and processes of lncRNAs in HCC remains limited.

**Methods:**

The expression level of USP27X‐AS1 was assessed in an HCC patient cohort through a combination of bioinformatics analysis and qRT‐PCR. Subsequent biological experiments were conducted to delve into the functional aspects of USP27X‐AS1. Additional molecular biology techniques, including RNA pulldown and RNA immunoprecipitation (RIP), were employed to elucidate the potential mechanisms involving USP27X‐AS1 in HCC. Finally, CUT–RUN assay and other investigations were carried out to determine the factors contributing to the heightened expression of USP27X‐AS1 in HCC.

**Results:**

High expression of the novel oncogene USP27X‐AS1 predicted poor prognosis in HCC patients. Further investigation confirmed that USP27X‐AS1 promoted the proliferation and metastasis of HCC by enabling USP7 to interact with AKT, which reduced level of AKT poly‐ubiquitylation and enhanced AKT protein stability, which improves protein stabilisation of AKT and promotes the progression of HCC. Moreover, we also revealed that SP1 binds to USP27X‐AS1 promoter to activate its transcription.

**Conclusions:**

Novel oncogenic lncRNA USP27X‐AS1 promoted HCC progression via recruiting USP7 to deubiquitinate AKT. SP1 transcriptionally activated USP27X‐AS1 expression. These findings shed light on HCC and pointed to USP27X‐AS1 as a potential predictive biomarker and treatment target for the malignancy.

## Graphical abstract

(1) First clarification of the biological role of USP27X‐AS1 in hepatocellular carcinoma (HCC).

(2) Expanding the mechanisms by which AKT promotes the development of HCC.

(3) Provides a potential therapeutic target for HCC.

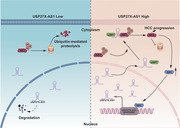

## 1 BACKGROUND

Hepatocellular carcinoma (HCC) stands as the sixth most prevalent malignant tumour globally and represents a major contributor to cancer‐related mortality.^1^ Despite the array of available treatment modalities for HCC, their efficacy remains suboptimal, necessitating the identification and development of new treatment targets for enhanced HCC management.

Non‐coding RNAs constitute a substantial portion, encompassing 90% of the human genome. Recent investigations have unveiled the functions of non‐coding RNAs, underscoring their pivotal roles in diseases.^2^ Long non‐coding RNA (lncRNA) is a member of the non‐coding RNAs family, consisting of 200 or more nucleotides.^3^ It actively participates in various cellular processes, including chromatin remodelling, gene transcription, post‐transcriptional regulation, protein localisation and intermolecular signalling pathways.^4^, ^5^, ^6^ In HCC, previous studies have shown that the lncRNA is involved in the development of cancer.^7^, ^8^, ^9^, ^10^ However, the precise mechanisms governing lncRNA functions in cancer demand elucidation. Consequently, a more comprehensive understanding of the role of lncRNA in HCC progression promises a fresh perspective for the diagnosis and treatment of this malignancy.

AKT is a serine and threonine kinase that plays a key role in cell‐to‐cell signalling pathway during tumorigenesis and tumour progression.^11^ AKT has numerous post‐translational modifications.^12^ The most famous one is phosphorylation, which is crucial for AKT kinase activation after membrane translocation.^13^, ^14^, ^15^ Besides, recent research has shown that AKT also has other post‐translation modifications, such as ubiquitination, acetylation, methylation, SUMOylation, and so forth.^16^, ^17^, ^18^, ^19^, ^20^, ^21^, ^22^, ^23^ At the level of ubiquitination, E3 ligase NEDD4‐1, TRAF6 and TRAF4 could drive K63‐linked ubiquitination of AKT, leading to AKT membrane translocation and subsequent phosphorylation.^20^, ^24^, ^25^ AKT also exhibits K48‐linked ubiquitination, leading to proteasome degradation. In Down syndrome (DS), E3 ligase TTC3 induces AKT ubiquitination to promote its degradation.^26^ In malignancies, E3 ligase MULAN and BRAC1 were reported to ubiquitinate p‐AKT for degradation to inhibit cancer progression.^16^, ^17^ However, like phosphorylation, ubiquitination is a reversible process. It has been reported that CYLD was responsible for the removal of K63‐linked ubiquitination of AKT in lung fibrosis.^27^ For the study of AKT ubiquitination, most focused on protein regulation, with little research undertaken into the regulation of lncRNAs.

Ubiquitin‐specific protease (USP7), also referred to as herpesvirus‐associated ubiquitin‐specific protease (HAUSP), is a 130‐kDa protein characterised by multiple domains facilitating interactions with a diverse array of proteins. These interactions play crucial roles in fundamental developmental and homeostatic processes, encompassing the cell cycle,^28^ immune response,^29^ as well as the modulation of transcription factor and epigenetic regulator activity and localisation.^30^, ^31^, ^32^ Furthermore, USP7 contributes to carcinogenesis by inappropriately activating the Wnt signalling pathway^33^, ^34^ and stabilising HIF‐1α.^34^ Collectively, these findings underscore the multifaceted involvement of USP7 in diverse biological functions throughout tumour progression.

In this study, we identified a novel lncRNA, USP27X‐AS1. By analysing online databases and our own clinical samples, we found that USP27X‐AS1 is upregulated in HCC, and is associated with poor prognosis. lncRNAs generally perform their biological functions by acting as RBPs, and further studies demonstrated that USP27X‐AS1 interacts with AKT and forms a complex by recruiting USP7 and can reduce AKT ubiquitination. Meanwhile, SP1, a downstream transcription factor of AKT, was also shown to bind to the promoter region of USP27X‐AS1 to activate its transcription, which creates a positive feedback loop. Our study enriches the mechanism of lncRNA action in HCC, expands the novel regulatory mechanisms of the AKT pathway and identifies potential reliable targets for the treatment of HCC.

## 2 METHODS

### 2.1 Databases and bioinformatics analysis

For survival and gene expression analysis, the expression profiling data and relevant clinical information were collected from the Cancer Genome Atlas Liver Hepatocellular Carcinoma (TCGA‐LIHC, https://portal.gdc.cancer.gov/), Gene Expression Omnibus (GEO, http://www.ncbi.nlm.nih.gov/geo) or RNA sequences. Subsequent transcriptome analysis was performed using R (Version 4.2.3). Differentially expressed genes between groups were identified using the package DESeq2 (Version 1.36.0). Gene Set Enrichment Analysis (GSEA) was implemented using Cluster‐Profiler (Version 4.6.2). The heatmap, pan‐cancer expression and chordal graph were plotted using R, whereas the relative expression, correlation and Kaplan–Meier survival analysis and plot were implemented using GraphPad Prism (Version 9.0).

### 2.2 Clinical samples

The clinical samples utilised in this study were provided by Tongji Hospital of Huazhong University of Science and Technology (HUST) in Wuhan, China, and the study protocol received approval from the HUST Ethics Committee at Tongji Hospital.

### 2.3 Cell culture

The China Center for Type Culture Collection (Wuhan, China) provided human HCC cell lines, including HLF, Hep3B, LM3, MHCC97H, HepG2 and PLC/PRF/5, along with the normal liver cell line THLE3. Additionally, the human embryonic kidney cell line 293T (HEK‐293T) was acquired from the Shanghai Branch Cell Bank of the Chinese Academy of Sciences (Shanghai, China). All cell lines were cultured in Dulbecco's modified Eagle's medium (DMEM) supplemented with 10% fetal bovine serum (Beijing Dingguo changsheng Biotechnology Co., Ltd) and maintained at 37°C in a humid incubator with 5% CO_2_. The cell lines underwent rigorous examination to confirm the absence of mycoplasma, and their authenticity was verified through short‐tandem repeat analysis.

### 2.4 Plasmid construction and establishment of stable cell lines

AuGCT (AuGCT DNA‐SYN Biotechnology Co., Ltd) produced the full‐length human USP27X‐AS1 cDNA (2304 bp), which was then cloned into the expression vector pcDNA3.1(+) from Add‐gene. According to the secondary structure of USP27X‐AS1 predicted by the RNA fold Web Server (http://rna.tbi.univie.ac.at/), a number of USP27X‐AS1 mutants could be created. The following were purchased from Add‐gene: human AKT (1443 bp), USP7 (3309 bp) and SP1 (2358 bp) cDNA were subcloned into the plasmid pcDNA3.1(+)‐3xFlag‐CMV (Sigma‐Aldrich). According to the domain structure of AKT predicted by the Uni‐Port (https://www.uniprot.org/), a number of AKT mutants were generated. The entire AKT cDNA sequence was adopted to verify each mutation. Human ubiquitin (Ub) cDNA was subcloned into plasmid pcDNA‐3.1(−)‐HA. Purinomycin was utilised to generate and evaluate a stable USP27X‐AS1‐overexpressing HLF and Hep3B cell line (Solarbio Life Science). The USP27X‐AS1 small hairpin RNA (shRNA) was donated by Sigma Corporation. Following the manufacturer's instructions, stable USP27X‐AS1‐knockdown MHCC97H and LM3 cells were created using a lentiviral vector. Tables S1–S3 contain a complete list of all primers and shRNA sequences. As an overexpression control (Con), non‐target (NT) shRNA was employed, and the empty vector pcDNA‐3.1(−)‐HA or the plasmid pcDNA3.1(+)‐3xFlag‐CMV was used as a knockdown control.

### 2.5 RNA extraction and quantitative real‐time PCR (RT‐qPCR)

Total RNAs were extracted from cells or tissues using the FastPure Cell/Tissue Total RNA Isolation Kit V2 (RC112‐01, Vazyme). Reverse transcription was carried out with the reverse transcription kit (R233‐01, Vazyme). For qPCR, the Universal SYBR qPCR Master Mix kit (Vazyme, Q711‐02) was employed.

### 2.6 Rapid amplification of cDNA ends

As instructed by the manufacturer, rapid amplification of cDNA ends (RACE) was carried out using the HiScript‐TS 5′/3′ RACE Kit (RA101‐01, Vazyme). Using the universal primers and the USP27X‐AS1‐specific primer pair, PCR was used to conduct the 5′ and 3′ RACE reactions. Sanger sequencing enabled identification of the amplified fragment.

### 2.7 CCK‐8 assay

HCC cells were cultured to logarithmic growth phase and subsequently digested using trypsin, resuspended and counted, and then at 1000 cells per well, inoculated into 96‐well plates with several replicate wells. Approximately 6−8 h were allowed for the cells to attach to the wall, that is, Day 0. A CCK‐8 Cell Counting Kit (A311‐01, Vazyme) was used to test the OD (450 nm) value over 5 days.

### 2.8 Colony formation assay

HCC cells were cultured to logarithmic growth phase and subsequently digested, resuspended and counted using trypsin, then inoculated into six‐well plates with three replicate wells at 1000 cells per well. We incubated the cells for 10–14 days (replacing the complete medium every 3 days); when cell clumps were visible to the naked eye, the specimens were fixed with 4% paraformaldehyde, then stained with crystal violet, a picture was taken with a camera and were counted with imageJ software.

### 2.9 EdU incorporation assay

A 24‐well plate was filled with cells, which was then incubated to a 40% density, and 100 µL of 50 M EdU resolution was added to the cells and treated for 2 h (BeyoClick EdU Cell Proliferation Kit with a Fluor 594, Beyotime, C0078S). Cells were then fixed with 4% paraformaldehyde after being rinsed with PBS. Cells were ruptured with .5% Triton X‐100 for 30 min, after which they were stained with 100 µL of 1X Apollo solution and 1 Hoechst 33342 solution before a fluorescence microscope was used to take pictures and ascertain cell counts.

### 2.10 Invasion and migration assays

Matrigel was diluted with DMEM at 1:4. We then added 50 µL diluted Matrigel into the upper chamber for invasion assay. Cells were seeded into upper chambers as follows: 250 000 cells for migration assay and 500 000 cells for invasion assay. Complete medium was added into the lower chambers. After 24–48 h, we washed the upper chamber with PBS, then fixed the specimen with 4% paraformaldehyde for 15 min and stained with crystal violet for 15 min, facilitating subsequent microscopy and counting.

### 2.11 Wound‐healing assay

Cells were cultured to logarithmic growth phase, subsequently digested using trypsin, resuspended and counted, and depending on the size of the cells, the appropriate number of cells were seeded into a six‐well plate to form a single dense cell layer. After the cells were fully walled, a pipette was used to scrape across the confluent cell layer to form a linear wound. DMEM was replaced with complete medium. Photographs were taken at 0, 24 and 48 h to observe the wound healing and the degree of healing was calculated using imageJ software.

### 2.12 Western blot assay

Human tissues or cells were lysed in RIPA buffer supplemented with Roche's phosphatase inhibitor cocktail and EDTA‐free protease inhibitor cocktail. Protein concentrations were determined using the bicinchoninic acid assay. Subsequently, cell and tissue lysates were subjected to 10% sodium dodecyl sulfate‐polyacrylamide gel electrophoresis and transferred onto polyvinylidene fluoride membranes. Following a block in 5% skimmed milk for 1 h, the membranes were pre‐incubated with the appropriate primary antibody overnight at 4°C. This was followed by incubation with the corresponding secondary antibodies for 1 h at room temperature. Immunoreactive bands were visualised using Bio‐Rad GelDoc technology and ClarityTM Western ECL substrate (both from Bio‐Rad). The antibodies utilised are detailed in Table S4.

### 2.13 Immunohistochemistry (ISH)

The ISH Kit (Boster, Bioengineering Company) was used to execute the procedure in accordance with manufacturer's instructions. Specimens were haematoxylin‐stained, alcohol‐dehydrated, xylene‐washed, and flavour‐sealing tablets were used thereon. For USP27X‐AS1, Oligo was employed as an ISH probe. Using a DM2300 microscope and ScopeImage 9.0 software (Nanjing Jiangnan Novel Optics Co., Ltd), representative images of ISH were taken and processed. Three pathologists independently determined the ISH staining scores without knowing the patient's history. The percentage of positive cells was multiplied by the staining intensity score, as previously mentioned to obtain the overall scores.

### 2.14 The CRISPR‐Cas9 system

Target DNA sequences that were introduced into Lenti‐V2‐puro for the USP7 exon 2 CRISPR‐cas9 mediated knockout SgRNA were created. According to the manufacturer's instructions, the plasmids were transfected into MHCC97H cells to produce a USP7 knockout (KO) cell line. Puromycin was applied to the cells, then they were divided into single cells to create colonies. These colonies were collected, and Western blotting and Sanger sequencing were used to confirm that they were USP7 KO.

### 2.15 Animal models

All the animals used in this study were 4‐week‐old, male, BALB/c nude mice. For subcutaneous tumour model, each mouse was injected with 1 000 000 cells/100 µL. Twenty‐eight days later, tumours were removed, fixed, weighed, photographed and stored. For the orthotopic tumour model, each mouse was injected with 1 000 000 cells/30 µL in the left lobe of liver. Some 28 days later, tumours were removed, fixed, weighed, photographed and stored. For the lung metastasis model, each mouse was injected with 1 000 000 cells/100 µL in the tail vein. Some 28 days later, tumours were removed, fixed, weighed, photographed and stored.

### 2.16 RNA‐fluorescence in situ hybridisation

Fluorescence‐attached USP27X‐AS1 probes were used in fluorescence in situ hybridisation (FISH) experiments. The USP27X‐AS1 protein was tagged with a cyanine 3‐conjugated RNA probe (red), and the nuclei were coloured blue by the addition of 4′,6‐diamidino‐2‐phenylindole. The Fluorescent In Situ Hybridisation Kit (RiboBio) was used for the FISH assay in accordance with the manufacturer's instructions. A Carl Zeiss AG, Jena, LSM 710 confocal microscope was used to capture imagery (micrographs).

### 2.17 RNA‑sequence analysis

HLF vector cells and USP27X‐AS1 overexpression cells were lysed with Trizol (Sigma). RNA extraction, library preparation, transcriptome sequencing and data analysis were conducted by the Haplox Genomics Center (Jiangxi, China).

### 2.18 RNA pulldown assay

Total RNA was extracted using the described method. Subsequently, 100‐nmol RNA probes were added and incubated at 70°C for 5 min before cooling to room temperature. Streptavidin magnetic beads were introduced and left to incubate for 1 h at room temperature on a shaking table. Afterward, we washed the magnetic beads with 20‐mM Tris to eliminate unbound RNA, followed by three washes with washing buffer. A subsequent incubation with 50 µL elution buffer for 1.5 h occurred on a vertical rotary shaker at 4°C. Next, specimens were incubated at 37°C for 15 min with stirring. The supernatant was collected, and lysates underwent Western blotting analysis using appropriate antibodies.

### 2.19 Mass spectrometry (MS)

The potential binding protein of USP27X‐AS1 was pulled down by RNA pulldown assay, and the supernatant was submitted to the SpecAlly (SpecAlly Life Technology Co., Ltd, Wuhan, China) for MS.

### 2.20 RNA‐binding protein immunoprecipitation (RIP)

The Millipore Magna RIPTM RNA‐Binding Protein Immunoprecipitation Kit was employed for RIP, adhering to the manufacturer's instructions. Specifically, 1 × 10^7^ HCC cells were collected and lysed with RIP lysis solution. The cell extract underwent treatment with magnetic beads coupled with antibodies against the Flag‐tag or unconjugated mouse immunoglobulin G (mIgG) as a reference. Subsequently, the beads were rinsed, and proteinase K incubation was followed to remove the proteins. Amplification of the recovered RNA was performed using USP27X‐AS1‐specific primers via qRT‐PCR. To validate the qRT‐PCR results, simultaneous measurements of total RNAs (used as input controls) and normal IgG controls were conducted.

### 2.21 Co‐immunoprecipitation (Co‐IP)

Cells were lysed with 400–800 µL IP lysis. Cell lysate and antibodies were incubated overnight on a vertical rotary shaker at 4°C. We then added washed protein A/G magnetic beads (HY‐K0202, MedChemExpress) into the supernatant, and incubated the specimens for 2–4 h on a vertical rotary shaker at 4°C. We discarded the supernatant, washed the magnetic beads with TBST five, discarded the TBST, added 50–100 µL 1 × loading buffer, and incubated the specimens in boiling water for 10 min. The precipitants and lysates were subjected to Western blotting analysis with appropriate antibodies.

### 2.22 Ubiquitination‐based Co‐IP

Cells were transfected with HA‐tagged ubiquitination; 48 h later, cells were lysed with IP lysis solution (subsequent steps were as described previously). The ubiquitination level was detected by incubating HA‐Tag antibody. The precipitants and lysates were subjected to Western blotting analysis with appropriate antibodies.

### 2.23 CUT&RUN

The Hyperactive pG‐MNase CUT&RUN Assay Kit for PCR/qPCR (Vazyme, HD101‐01) was used to conduct the CUT&RUN assay. Some 1 × 10^5^ HCC cells were gathered, washed once in 100 µL of washing buffer, then bound to ConA beads for 10 min at 25°C. Cells were then treated with 1 µL of SP1 (CST, #9389) antibody at 4°C overnight. The following day, pG‐MNase enzyme was added, and the cells were incubated for 1 h at 4°C after being washed twice with DIG washing buffer. Following two rounds of DIG washing, cells were incubated with CaCl_2_ for 1 h at 4°C. We added 100 mL of stop buffer, resuspended the cells, and incubated the specimens for 30 min at 37°C. Cells were plated on a magnet after incubation, and any unattached liquid was drained. DNA was eluted from the beads with double‐distilled water after they had been gently washed twice with 80% ethanol. qRT‐PCR tests were conducted in accordance with the manufacturer's recommendations.

### 2.24 Luciferase reporter assay

The SP1‐promoter sequence was cloned onto the pGL4.17 vector. Cells were seeded into a 24‐well plate at 40% density, then transfected with 200 ng pGL4.17 plasmid and 4 ng pRL‐TK‐Renilla‐luciferase plasmid; 48 h later, cells were collected with Passive Lysis Buffer (Promega) according to the instructions. The next steps were performed according to the protocol. The luciferase activity was measured by GloMax 20/20 luminometer (Promega). The relative luciferase activity was determined based on the ratio of firefly luciferase to Renilla luciferase.

### 2.25 Statistical analysis

The data were presented as mean ± standard deviation. Statistical analyses were performed using GraphPad Prism 9.0 (GraphPad Software) and SPSS 20.0 (SPSS Inc.), and significance was defined as *p* < .05.

## 3 RESULTS

### 3.1 Elevated expression of USP27X‐AS1 correlated with poor prognosis in patients with HCC

**FIGURE 1:**
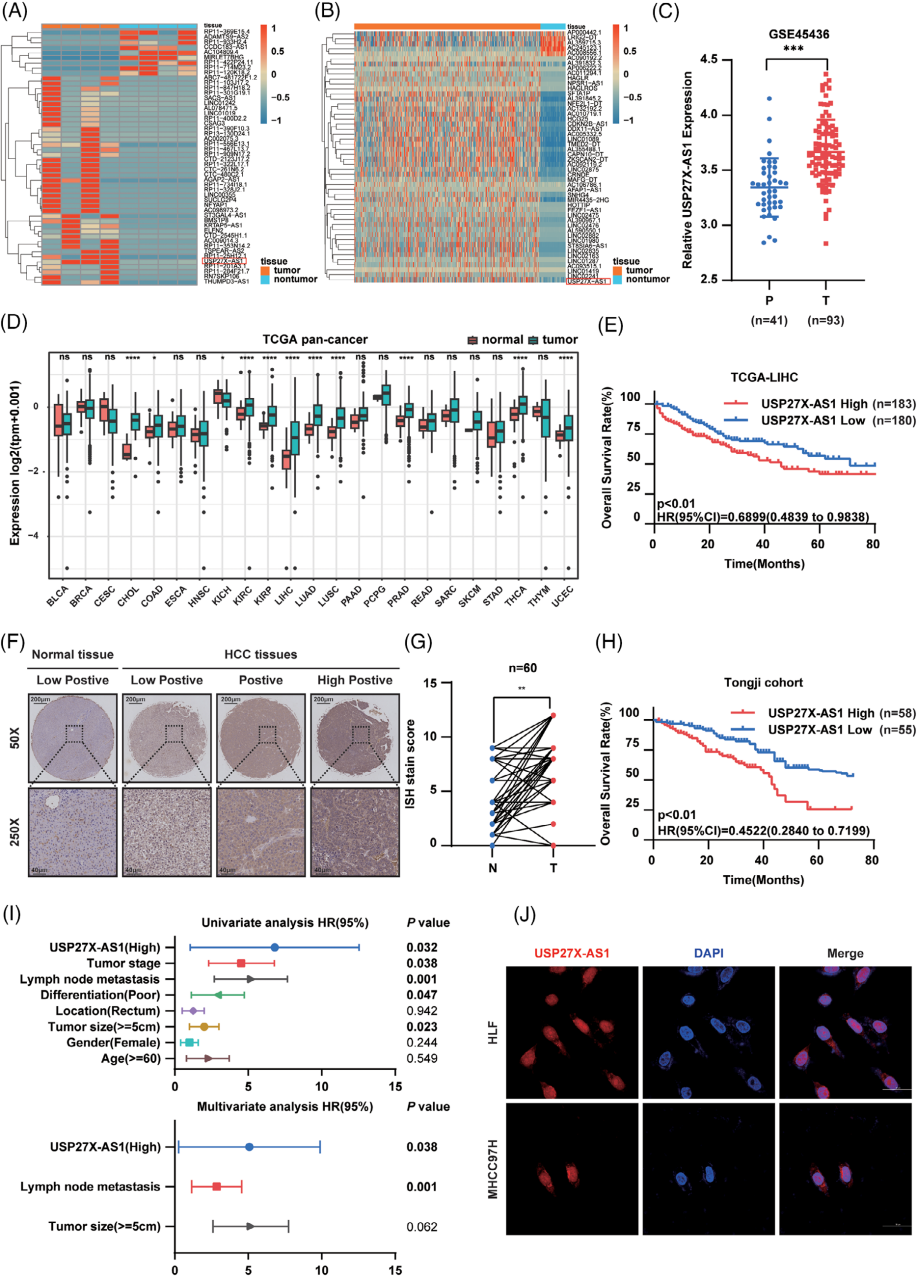
Elevated expression of USP27X‐AS1 correlated with adverse clinical outcomes in patients with hepatocellular carcinoma (HCC). (A and B) Heatmap of significant differentially expressed lncRNAs in the RNA‐seq from Tongji samples (A) and TCGA LIHC database (B) (USP27X‐AS1 is highlighted in red box). (C) The expression of USP27X‐AS1 in HCC cohort GSE45436. (D) The expression of USP27X‐AS1 in pan‐cancer. (E) Kaplan–Meier survival analyses of overall survival (OS) based on USP27X‐AS1 expression in TCGA LIHC database. (F) Representative images of USP27X‐AS1 ISH from normal tissues and HCC tissues. Scale bar: 200 µm (up), 40 µm (bottom). (G) ISH score of USP27X‐AS1 from Tongji cohort, *n* = 60. (H) Kaplan–Meier survival analyses of OS rate based on USP27X‐AS1 expression from Tongji cohort. (I) Univariate and multivariate regression analyses of HCC patients from Tongji cohort. (J) Identification of the subcellular localisation of USP27X‐AS1 by FISH. Data and error bars are shown as mean ± SD of triplicate independent replicate experiments. *p < .05, **p < .01, ***p < .001, ns: no significance. Data were analysed by paired Student's *t*‐test (C and G). Log‐rank test was used for survival comparison (E and H).

We conducted transcriptional profiling analyses, employing next‐generation sequencing, on four paired HCC tissue specimens along with adjacent non‐tumour tissue specimens from the Tongji cohort to identify potential HCC‐related lncRNAs. (Figure 1A). TCGA database was also used to identify the top 50 differentially expressed lncRNAs (Figure 1B). Significantly, USP27X‐AS1 was upregulated in both cohorts. The abnormal expression of USP27X‐AS1 was also validated in five additional HCC cohorts GSE45436, GSE94660, GSE62232, GSE84402 and GSE87630 (Figure 1C and Figure S1A–D). Moreover, pan‐cancer data analysis showed that USP27X‐AS1 was upregulated in a variety of cancers including HCC, suggesting it was related to cancer biology (Figure 1D). Kaplan–Meier survival analysis indicated that a high level of USP27X‐AS1 was correlated with poor overall survival (OS) in the TCGA LIHC cohort. (Figure 1E). Online tools were used to confirm that USP27X‐AS1 was a conserved, non‐coding gene (Figure S1E–G). 5´3´ RACE assay showed that the full‐length of USP27X‐AS1 was 2308 bp (Figure S2A). To confirm the expression of USP27X‐AS1 in HCC, we examined its expression in an HCC tissue microarray from the Tongji cohort. As expected, USP27X‐AS1 was upregulated in HCC tissues and associated with poor OS and progression‐free survival (PFS) rate (Figure 1F–H and Figure S2B, Table S5). Moreover, quantitative reverse transcription‐polymerase chain reaction (qRT‐PCR) revealed upregulation of USP27X‐AS1 in an additional 40 pairs of HCC tissue specimens and their corresponding adjacent non‐tumour tissues (Figure S2C,D). Additionally, both univariate and multivariate Cox proportional hazard analyses demonstrated that USP27X‐AS1 served as an independent prognostic factor for HCC (Figure 1I and Table S6). Collectively, these findings suggest that the novel lncRNA USP27X‐AS1 is upregulated in HCC and predicts an unfavourable prognosis. Next, we used confocal microscopy analysis of fluorescent in situ hybridisation and nuclear/cytoplasm fractionation to ascertain the subcellular localisation of USP27X‐AS1. The findings revealed dual localisation of USP27X‐AS1 in both the cytoplasm and nucleus, with over half of the molecules situated in the cytoplasm (Figure 1J and Figure S2E–H).

### 3.2 USP27X‐AS1 promoted HCC progression in vitro

**FIGURE 2:**
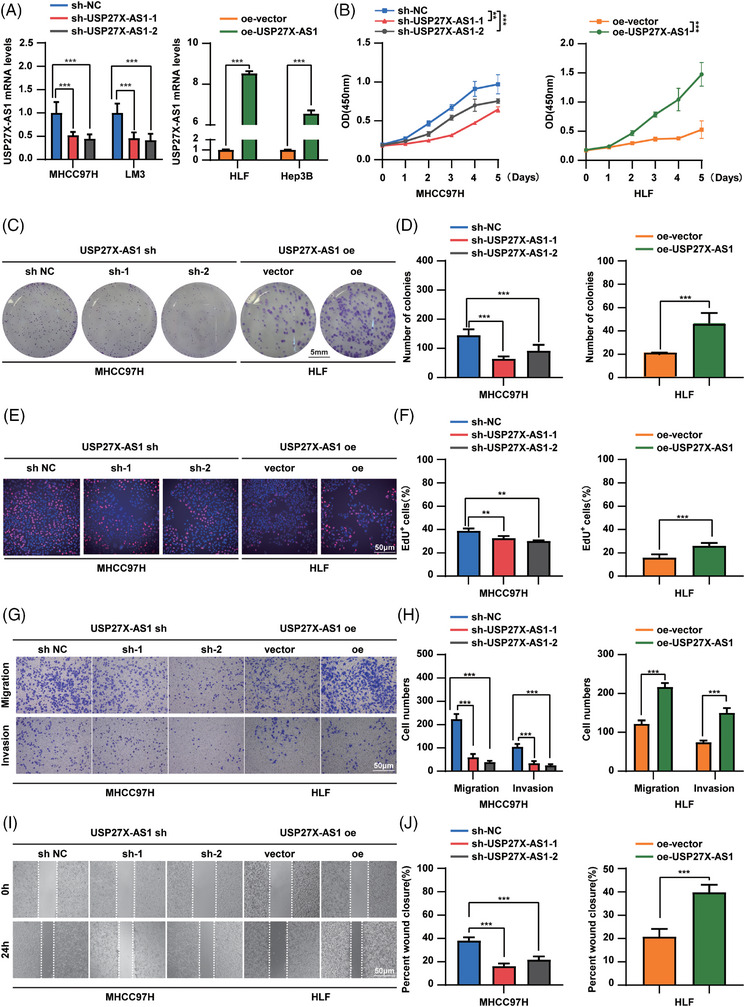
USP27X‐AS1 promoted hepatocellular carcinoma (HCC) proliferation, migration and invasion in vitro. (A) qRT‐PCR tested the efficacy of knockdown (left) or overexpression (right) of USP27X‐AS1 in HCC cell lines. (B) CCK‐8 assay of MHCC97H USP27X‐AS1 knockdown cell line and HLF USP27X‐AS1 overexpression cell line. (C and D) Representative images (C) and number (D) of colonies in MHCC97H USP27X‐AS1 knockdown cell line and HLF USP27X‐AS1 overexpression cell line. (E and F) Representative images (E) and positive cell number (F) of EdU assay in MHCC97H USP27X‐AS1 knockdown cell line and HLF USP27X‐AS1 overexpression cell line. (G and H) Representative images (G) and number (H) of migration or invasion cells in MHCC97H USP27X‐AS1 knockdown cell line and HLF USP27X‐AS1 overexpression cell line. (I and J) Representative images (I) and wound closure rate (J) of wound healing assay in MHCC97H USP27X‐AS1 knockdown cell line and HLF USP27X‐AS1 overexpression cell line. Data and error bars are shown as mean ± SD of triplicate independent replicate experiments. *p < .05, **p < .01, ***p < .001, ns: no significance. Data were analysed by paired Student's *t*‐test (A, B, D, F, H and J).

To investigate this hypothesis, we generated cell lines with overexpression or knockdown of USP27X‐AS1 in MHCC97H, LM3, HLF and Hep3B cell lines, resulting in the relative upregulation or downregulation of USP27X‐AS1 (Figure 2A and Figure S3A). Cellular viability and colony‐forming potential were assessed using CCK‐8 and colony formation assays, revealing that USP27X‐AS1 overexpression significantly enhanced these parameters in HLF and Hep3B cells (Figure 2B–D and Figure S3B–D). Conversely, USP27X‐AS1 knockdown reduced cell viability and colony‐formation ability in MHCC97H and LM3 cells (Figure 2B–D and Figure S3B–D). EdU cell proliferation assay further demonstrated an increased percentage of S‐phase cells in USP27X‐AS1 overexpressing cells (Figure 2E,F and Figure S3E,F), while knockdown of USP27X‐AS1 decreased the population of EdU‐positive cells in MHCC97H and LM3 cells (Figure 2E,F and Figure S3E,F). Transwell and wound‐healing assays indicated that USP27X‐AS1 overexpression enhanced the migration and invasion abilities of HLF and Hep3B cells (Figure 2G–J and Figure S3G–J), whereas USP27X‐AS1 knockdown diminished these capabilities in MHCC97H and LM3 cells (Figure 2G–J and Figure S3G–J). All these results confirmed the HCC‐promoting ability of USP27X‐AS1.

### 3.3 USP27X‐AS1 promoted HCC progression in vivo

**FIGURE 3:**
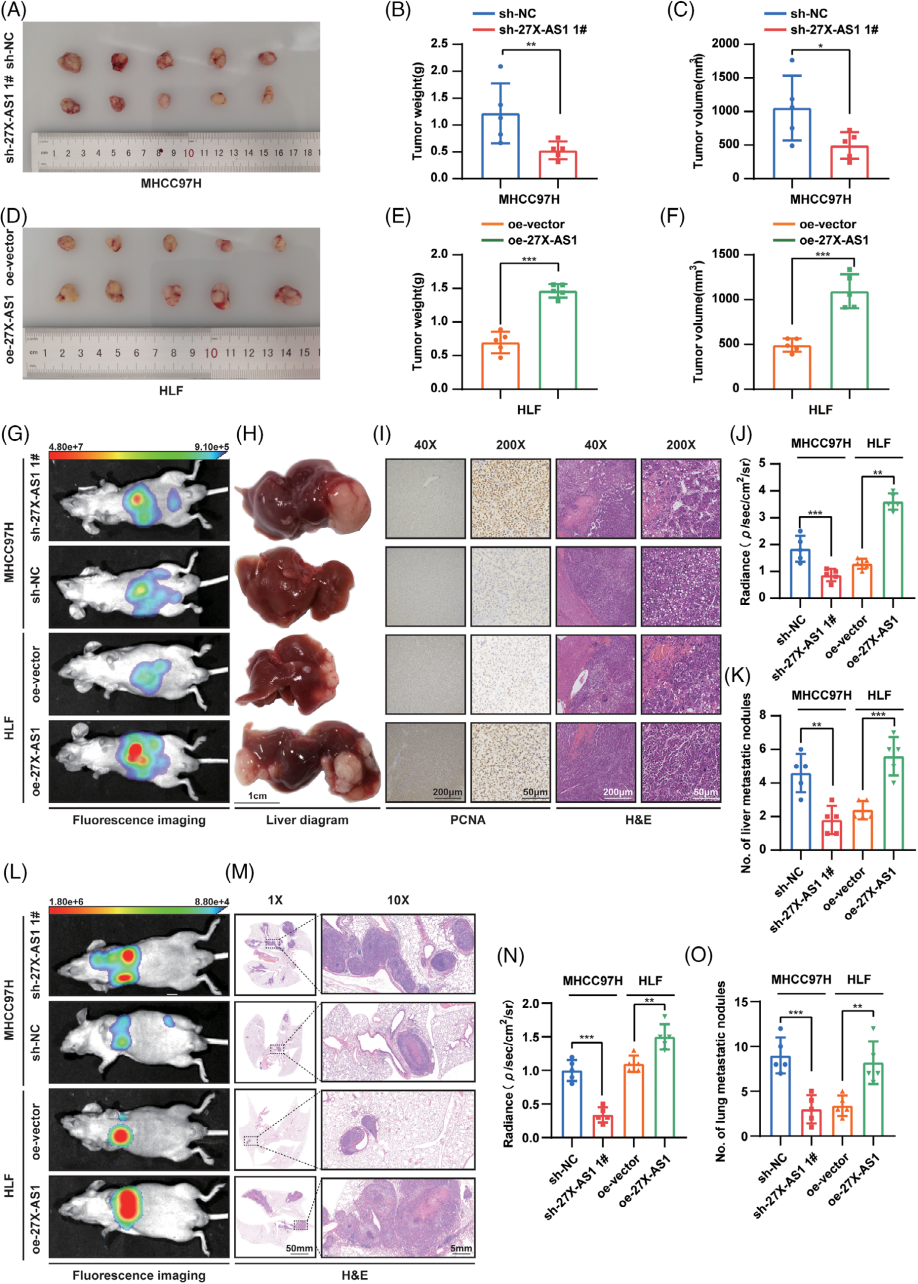
USP27X‐AS1 promoted hepatocellular carcinoma (HCC) proliferation, migration and invasion in vivo. (A–C) Gross image (A), tumour weight (B), tumour volume (C) from control group and USP27X‐AS1 knockdown group in subcutaneous tumour model. (D–F) Gross image (D), tumour weight (E), tumour volume (F) from control group and USP27X‐AS1 overexpression group in orthotopic model. (G–K) Representative fluorescence images (G), representative liver diagram (H), representative PCNA and HE images (I), radiance (J), number of liver metastasis nodules (K) from shNC, shUSP27X‐AS1, oe‐vector and oe‐USP27X‐AS1 group; scale bar: 1 cm (H), 200 µm (40×), 50 µm (200×). (L–O) Representative fluorescence images (L), representative HE images (M), radiance (N), number of lung metastasis nodules (O) from shNC, shUSP27X‐AS1, oe‐vector and oe‐USP27X‐AS1 group in tail vein injection metastasis model; scale bar: 50 mm (1×), 5 mm (10×). Data and error bars are shown as mean ± SD of triplicate independent replicate experiments. *p < .05, **p < .01, ***p < .001, ns: no significance. Data were analysed by paired Student's *t*‐test (B, C, E, F, J, K, N and O).

Next, we validated the oncogenic role of USP27X‐AS1 in vivo. Subcutaneous tumour model showed that knockdown of USP27X‐AS1 decreased tumour volume and tumour weight compared to the control group (each group *n* = 5) (Figure 3A–C). While overexpression of USP27X‐AS1 showed a greater tumour burden (Figure 3D–F). Moreover, the orthotopic model indicated that knockdown of USP27X‐AS1 led to a lower tumour burden, lower PCNA and impaired intrahepatic metastasis ability compared to control (Figure 3G–K). Instead, overexpression of USP27X‐AS1 increased tumour burden, PCNA and liver metastasis nodules (each group *n* = 7) (Figure 3G–K). We then injected USP27X‐AS1 knockdown or overexpression cells into nude mice teil vein. Four weeks later, all groups developed lung metastatic tumours. However, the USP27X‐AS1 knockdown group had fewer (and smaller) metastatic tumours than the control group (Figure 3L–O). The number and volume of metastatic tumours in the USP27X‐AS1 overexpression group were higher than those in the control group (each group *n* = 7) (Figure 3L–O). The obtained results conclusively verify that USP27X‐AS1 promotes both cell proliferation and metastasis of HCC in vivo.

### 3.4 USP27X‐AS1 regulated the PI3K‐AKT signalling pathway

**FIGURE 4:**
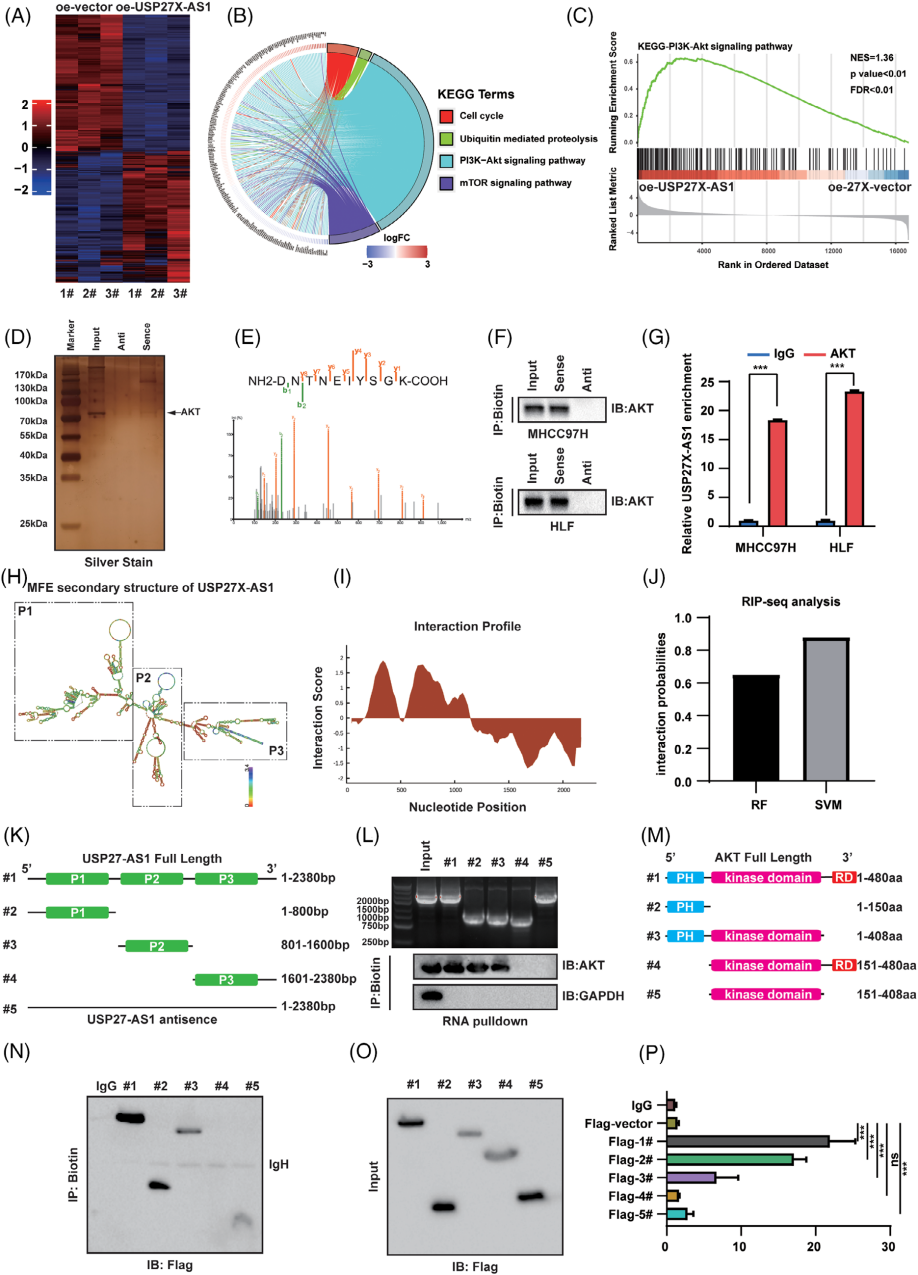
USP27X‐AS1 upregulated PI3K‐AKT pathway and interacted with AKT. (A) Heatmap of the differentially expressed genes between USP27X‐AS1 overexpression and control cells (*n* = 3). (B) The top four KEGG pathway terms significantly enriched in the USP27X‐AS1 overexpression regulated genes (*n* = 3). (C) GSEA plots of PI3K‐AKT pathway signatures in USP27X‐AS1 overexpression cells versus control cells (*n* = 3). (D) Silver stain of proteins enriched by USP27X‐AS1. (E) AKT peptides identified by USP27X‐AS1 MS. (F and G) RNA pulldown assay (F) and RIP assay (G) tested the interaction between USP27X‐AS1 and AKT in MHCC97H and HLF cells. (H) The predicted secondary structure of USP27X‐AS1. (I and J) Interaction profile of USP27X‐AS1 and AKT. (K) Diagram of truncated USP27X‐AS1. (L) RNA pulldown assay detected the interaction region of USP27X‐AS1 to bind AKT. (M) Diagram of truncated AKT. (N) RNA pulldown assay detected the interaction region of AKT to bind USP27X‐AS1. (O and P) RIP assay detected the interaction region of AKT to bind USP27X‐AS1. Data and error bars are shown as mean ± SD of triplicate independent replicate experiments. *p < .05, **p < .01, ***p < .001, ns: no significance. Data were analysed by paired Student's *t*‐test (G and P).

To unravel the mechanism by which USP27X‐AS1 promotes HCC, we conducted RNA‐seq on HLF cells with USP27X‐AS1 overexpression and the control counterparts. KEGG and GSEA analyses of the differentially expressed genes revealed that the PI3K‐AKT signalling pathway was the most enriched, and its activation positively correlated with USP27X‐AS1 expression (Figure 4A–C). Subsequently, we assessed AKT mRNA and protein expression in cell lines with USP27X‐AS1 overexpression or knockdown. The results demonstrated that USP27X‐AS1 had no impact on AKT mRNA expression, but showed a positive correlation with AKT protein expression (Figure S4A–H). Thus, we wondered whether USP27X‐AS1 regulated PI3K‐AKT signalling via post‐translational modification. As expected, mass spectrometry and silver stain indicated that USP27X‐AS1 enriched AKT protein (Figure 4D,E). RNA pulldown assay further confirmed that AKT interacted with USP27X‐AS1, but not UPS27X‐AS1 antisense (Figure 4F). RIP assay showed that AKT interacted with USP27X‐AS1 in HCC cell lines (Figure 4G and Figure S4I). All these results confirmed the interaction of USP27X‐AS1 and AKT. To determine the regions of USP27X‐AS1 to bind AKT, a series of USP27X‐AS1 deletion mutants could be constructed according to the predicted secondary structure of USP27X‐AS1 (Figure 4H–K). The CatRAPID on‐line algorithm was used to predict that the N‐terminus of USP27X‐AS1 was mainly responsible for the interaction of AKT (Figure 4I,J). RNA pulldown assay confirmed the responsible region of USP27X‐AS1 to bind AKT was between 1 and 1600 bp (Figure 4L). Truncated plasmids of AKT were constructed based on the structural domain (Figure 4M). RNA pulldown assay and RIP assay all indicated that the PH domain of AKT was responsible for the interaction (Figure 4N–P). Taken together, USP27X‐AS1 upregulated AKT signalling via interacting with AKT protein.

### 3.5 USP27X‐AS1 inhibited AKT proteasome degradation

**FIGURE 5:**
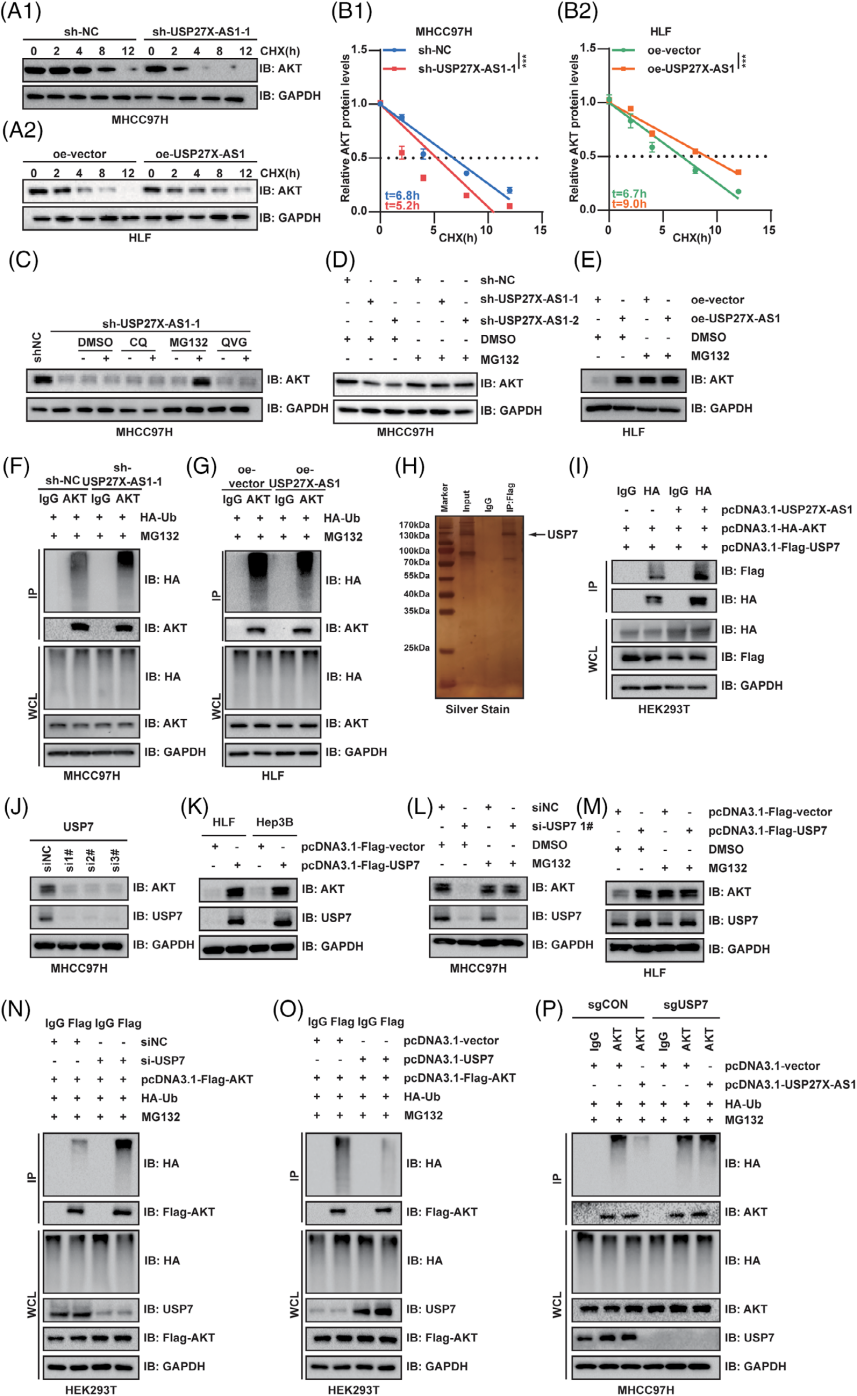
USP27X‐AS1 recruited USP7 to deubiquitinate AKT. (A and B) Half‐life assay tested the change of AKT stability under USP27X‐AS1 knockdown (MHCC97H) or overexpression (HLF) conditions. (C) Changes of AKT protein expression after DMSO (2 µL), CQ (10 µM), MG132 (10 µM) or QVG (10 µM) treatment 6 h in MHCC97H USP27X‐AS1 knockdown cells. (D and E) Changes of AKT protein expression in MHCC97H USP27X‐AS1 knockdown or HLF USP27X‐AS1 overexpression cells after treating with MG132 (10 µM) for 6 h. (F and G) Ubiquitination‐based endogenous Co‐IP detected the changes of AKT total poly‐ubiquitination level upon USP27X‐AS1 knockdown (MHCC97H) or overexpression (HLF). (H) Silver stain of proteins enriched by USP27X‐AS1. (I) Exogenous Co‐IP tested the interaction between USP7 and AKT with or without USP27X‐AS1 overexpression. (J) Changes of AKT protein expression after interference with USP7 expression in MHCC97H cells. (K) Changes of AKT protein expression after transfected HLF or Hep3B cells with Flag‐vector or Flag‐USP7. (L and M) Changes of AKT protein expression in MHCC97H interfered with USP27X‐AS1 or HLF USP27X‐AS1 overexpressed cells after treating with MG132 10 µM) for 6 h. (N and O) Ubiquitination‐based endogenous Co‐IP detected the changes of AKT total poly‐ubiquitination level upon USP27X‐AS1 interfered down‐ or overexpression. (P) Ubiquitination‐based endogenous Co‐IP detected the effect of USP27X‐AS1 on AKT poly‐ubiquitination level with or without USP7 knockout. Data and error bars are shown as mean ± SD of triplicate independent replicate experiments. *p < .05, **p < .01, ***p < .001, ns: no significance. Data were analysed by paired Student's *t*‐test (B).

The above results indicated that USP27X‐AS1 interacted with AKT and increased its protein expression. Next, we validated the stability of AKT upon CHX treating in USP27X‐AS1 knockdown or overexpression cells. Results showed that USP27X‐AS1 knockdown impaired AKT protein stability (Figure 5A1,B1 and Figure S5A1,B1), while USP27X‐AS1 overexpression prolonged the half‐life of AKT (Figure 5A2,B2 and Figure S5A2,B2), suggesting that USP27X‐AS1 might inhibit AKT degradation. There are two main degradation pathways in eukaryotic cells: lysosome degradation and proteasome degradation. To identify the degradation pathway through which USP27X‐AS1 affected AKT, we treated USP27X‐AS1 knockdown cells with MG132 (proteasome inhibitor), CQ (lysosome inhibitor) and QVG (caspase inhibitor). Results showed that only MG132 could effectively restore the protein level of AKT decreased by USP27X‐AS1 knockdown (Figure 5C and Figure S5C). Next, we further validated this result. After treating USP27X‐AS1 knockdown or overexpression cells with MG132, AKT protein level only restored in knockdown cell lines, indicating USP27X‐AS1 inhibited AKT proteasome degradation (Figure 5D,E and Figure S5D,E). Ubiquitination‐based Co‐IP analysis showed that knockdown of USP27X‐AS1 increased the total poly‐ubiquitination of AKT, and vice versa (Figure 5F,G and Figure S5F–I).

However, USP27X‐AS1 did not possess a DUBs function, so it might decrease AKT poly‐ubiquitination via recruiting DUBs to bind AKT. To prove this hypothesis, we identified the DUB USP7 from previous mass spectrometry (Figure 5H). Exogenous Co‐IP and endogenous Co‐IP showed that USP7 interacted with AKT, and the interaction of these two proteins was strengthened upon USP27X‐AS1 overexpression (Figure 5I and Figure S6G,H). Next, whether USP27X‐AS1 recruited USP7 to deubiquitinate AKT was verified; an RNA pulldown assay confirmed the interaction between USP27X‐AS1 and USP7 and showed that the 801−1600 bp region of USP27X‐AS1 was responsible for the binding to USP7 (Figure S6A,B). Based on the previous results, we speculated that AKT and USP7 bound to the N‐terminus (1–1600 bp) of USP27X‐AS1, bringing USP7 and AKT closer and facilitating their mutual interaction. Western blot analysis suggested that USP27X‐AS1 did not affect USP7 protein expression (Figure S6C–F), indicating that USP27X‐AS1 did not regulate AKT ubiquitination by affecting the expression of USP7. All these results confirmed that USP27X‐AS1 recruited USP7 to bind AKT.

Furthermore, decreasing USP7 expression using siRNAs downregulated AKT protein expression (Figure 5J and Figure S6I), while overexpression of USP7 increased AKT protein expression (Figure 5K). Half‐life analysis indicated USP7 stabilised AKT protein expression (Figure S7A,B). Moreover, USP7 downregulated‐mediated AKT decreasing could be restored by MG132 (Figure 5L,M and Figure S6J,K). Ubiquitination‐based Co‐IP showed that USP7 had the ability to decrease the total poly‐ubiquitination level of AKT (Figure 5N,O), further proving that USP7 inhibited AKT proteasome degradation via deubiquitinated AKT. Western blot analysis showed that USP27X‐AS1 could not upregulate AKT protein expression and poly‐ubiquitination level upon USP7 deficiency (Figure 5P and Figure S6L,M). Furthermore, USP27X‐AS1 had no effect on the stability of AKT protein expression when USP7 knockout occurred (Figure S7C,D). In conclusion, USP27X‐AS1 upregulated AKT protein expression by recruiting USP7 to deubiquitinate AKT.

### 3.6 USP27X‐AS1 promoted HCC progression by regulating AKT

**FIGURE 6:**
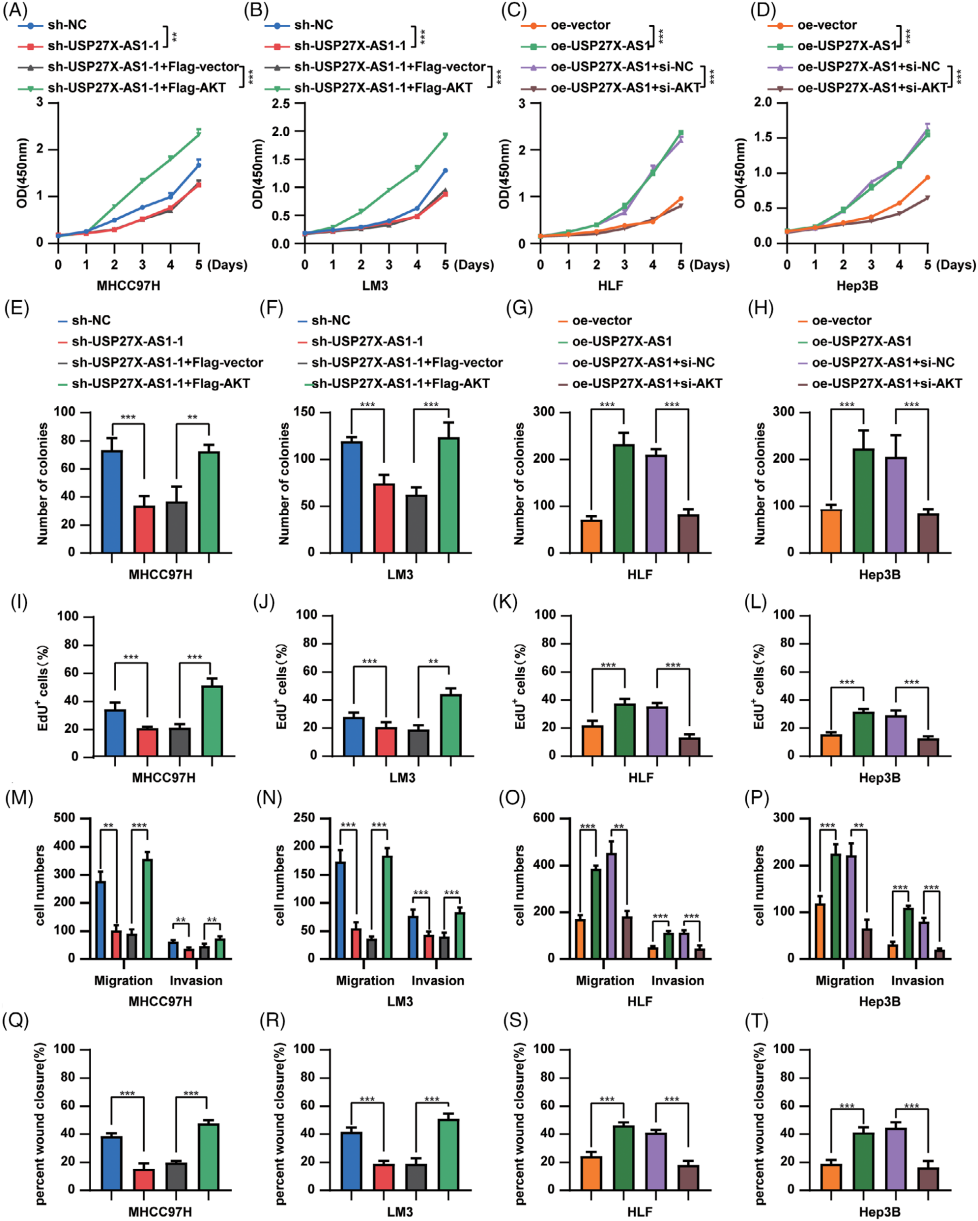
USP27X‐AS1 promoted hepatocellular carcinoma (HCC) progression by AKT. (A–D) CCK‐8 detected the effect of USP27X‐AS1 knockdown (MHCC97H, LM3) or overexpression (HLF, Hep3B) on the proliferation of HCC cells. (E–H) The number of colonies of the above‐mentioned cell lines. (I–L) The number of EdU^+^ cells of the above‐mentioned cell lines. (M–P) The number of migration and invasion cells of the above‐mentioned cell lines. (Q–T) The percentage of wound closure of the above‐mentioned cell lines. Data and error bars are shown as mean ± SD of triplicate independent replicate experiments. *p < .05, **p < .01, ***p < .001, ns: no significance. Data were analysed by paired Student's *t*‐test (A–T).

To verify whether USP27X‐AS1 promoted HCC progression via AKT, we transfected Flag‐AKT into USP27X‐AS1 knockdown cells and used AKT‐siRNA to interfere with AKT expression in USP27X‐AS1 overexpressed cells. qRT‐PCR and Western blot analysis confirmed the effectiveness of the transfection (Figure S8A–H). CCK‐8 assay, EdU assay and colony‐formation assay showed that overexpression of AKT could reverse the inhibition of HCC proliferation caused by decreasing USP27X‐AS1 (Figure 6A,B,E,F,I,J and Figure S8I,K), while USP27X‐AS1 lost the ability to promote HCC proliferation upon decreasing AKT (Figure 6C,D,G,H,K,L and Figure S8J,L). Transwell assay and wound‐healing assay indicated that the impaired migration and invasion ability caused by USP27X‐AS1 knockdown could be reversed by AKT overexpression (Figure 6M,N,Q,R and Figure S9A,C). Silencing of AKT eliminated the promoter functions of USP27X‐AS1, including migration and invasion (Figure 6O,P,S,T and Figure S9B,D). These data proved that USP27X‐AS1 promoted HCC progression via the AKT signalling pathway.

### 3.7 SP1 upregulated USP27X‐AS1 transcription

**FIGURE 7:**
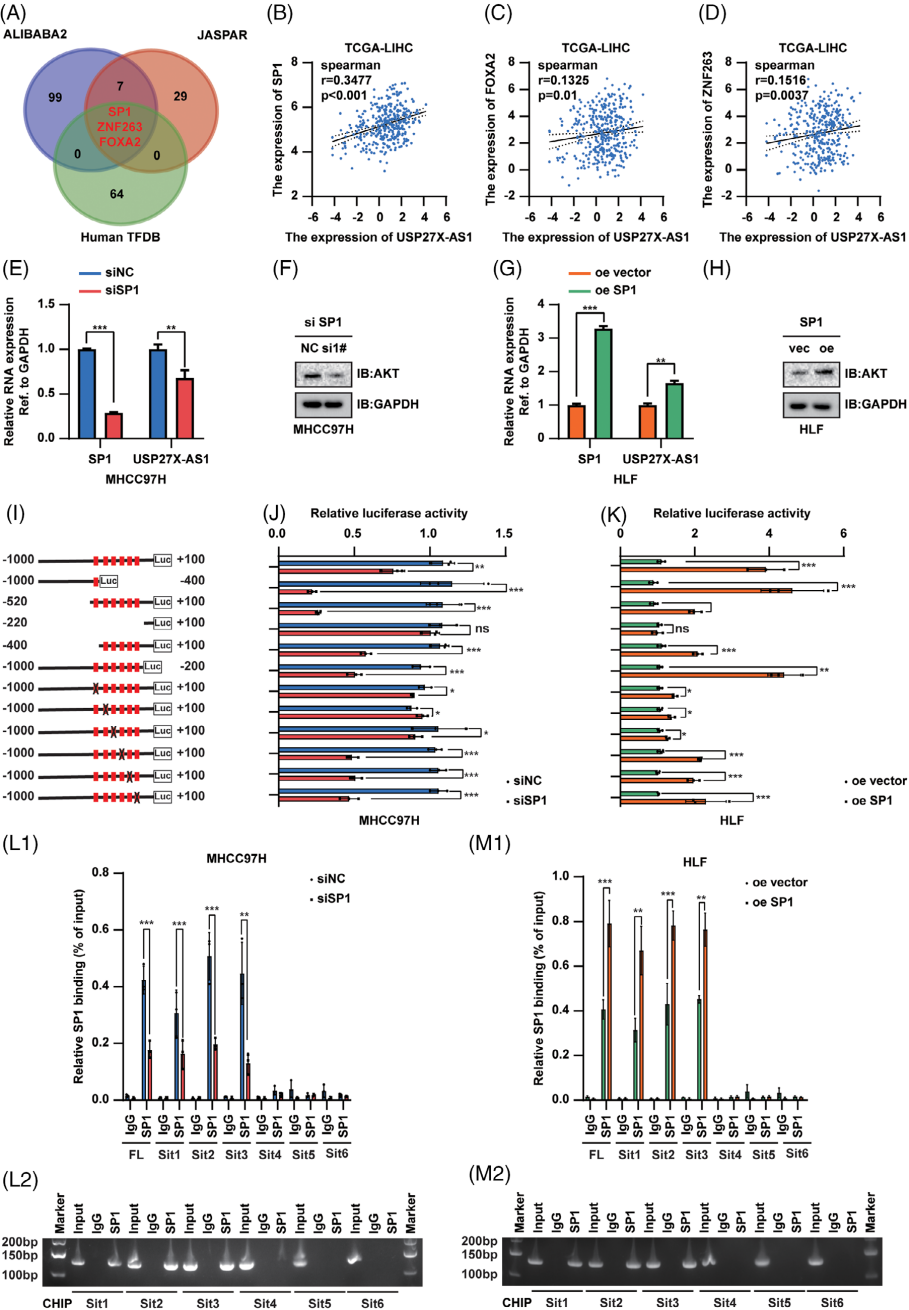
SP1 transcriptional upregulated USP27X‐AS1 expression. (A) Possible transcription factors of USP27X‐AS1 predicted by online tools. (B) Analyses for the expression correlations between SP1 and USP27X‐AS1 in TCGA‐LIHC; *r* = .3477, *p* < .001. (C) Analyses for the expression correlations between FOXA2 and USP27X‐AS1 in TCGA‐LIHC; *r* = .1325, *p* = .01. (D) Analyses for the expression correlations between ZNF263 and USP27X‐AS1 in TCGA‐LIHC; *r* = .1516, *p* = .0037. (E) Changes of SP1 and USP27X‐AS1 RNA level after SP1 expression was interfered in MHCC97H. (F) Changes of AKT protein level after SP1 expression was interfered in MHCC97H. (G) Changes of SP1 and USP27X‐AS1 RNA level after SP1 overexpression in HLF. (H) Changes of AKT protein level after SP1 overexpression in HLF. (I) Schematic diagram of the deletion and selective mutation of USP27X‐AS1 promoter. (J and K) Analysis identified SP1‐responsive regions in the USP27X‐AS1 promoter. (L and M) CUT–RUN analysis of SP1 binding to the USP27X‐AS1 promoter in MHCC97H (L) and HLF (M) cells. Data and error bars are shown as mean ± SD of triplicate independent replicate experiments. *p < .05, **p < .01, ***p < .001, ns: no significance. Data were analysed by paired Student's *t*‐test (B–E, G and J–M).

To further investigate the mechanism of USP27X‐AS1 overexpression in HCC, we used on‐line databases to seek transcription factors of USP27X‐AS1. After taking the intersection of three databases, three potential transcription factors were identified, including SP1, ZNF263 and FOXA2 (Figure 7A). We further verified the correlation between those three transcription factors and USP27X‐AS1 in TCGA LIHC database. The results showed that among these three transcription factors, SP1 had the strongest correlation with USP27X‐AS1 (Figure 7B–D). We then focused on SP1: qRT‐PCR and Western blot analysis showed that siSP1 decreased USP27X‐AS1 expression, and vice versa (Figure 7E–H and Figure S10A–D). Furthermore, potential SP1‐binding sites in the USP27X‐AS1 promoter were predicted using Jaspar (2020). Six putative binding sites were observed in the genomic region (Figure S10E,F). Luciferase assay combined with site‐deletion or site‐directed mutagenesis suggested that the binding sites 1, 2 and 3 induced SP1‐enhanced promoter activity in MHCC97H SP1‐overexpression cells (Figure 7I,J). CUT–RUN assay results also indicated that SP1 was recruited only to promoter regions containing binding sites 1, 2 and 3 in MHCC97H SP1 overexpression cells (Figure 7L). Similar results were also obtained by luciferase assay and CUT–RUN assay of HLF cells with SP1 knockdown (Figure 7K,M). Thus, binding sites 1, 2 and 3 were deemed critical to activating USP27X‐AS1 transcription by SP1.

## 4 DISCUSSION

In our present investigation, we discovered an emerging lncRNA, USP27X‐AS1, exhibiting elevated expression across various cancer types. Through extensive analysis of multiple HCC cohorts, we identified USP27X‐AS1 as a promising prognostic biomarker for HCC. Patients with higher USP27X‐AS1 levels experienced significantly shorter OS and PFS rates. However, the precise functions and mechanisms of USP27X‐AS1 in HCC remain undisclosed. The PI3K‐AKT signalling pathway plays a pivotal role in various biological processes in tumours, encompassing angiogenesis, cell cycle regulation, apoptosis, metabolism and cell proliferation.^35^, ^36^ While previous investigations into the activation of the PI3K‐AKT pathway have predominantly focused on AKT phosphorylation,^37^, ^38^, ^39^, ^40^, ^41^ some studies have explored the regulatory impact of proteins on AKT ubiquitination.^42^, ^43^, ^44^, ^45^ Our study introduces a novel finding, demonstrating that lncRNA USP27X‐AS1 also regulates the post‐translational modification of AKT. This discovery contributes fresh perspectives to the understanding of lncRNA involvement in the regulation of the AKT pathway.

Deubiquitylases have been implicated with a number of cancers, including HCC, according to recent research. USP7 regulates the function and turnover of a wide range of substrates, including P53, ERK1/2, PD‐L1, PRMT5, ANXA1, STAT3 and ECT2, which are crucial components of numerous oncogenic signalling pathways.^46^, ^47^, ^48^, ^49^, ^50^, ^51^, ^52^ For example, USP7 is a crucial deubiquitinase needed to stabilise oncogenic versions of DDX3X. By stabilising DDX3X, USP7 increases Wnt/beta‐catenin signalling, which has previously been demonstrated to be strongly correlated with colorectal cancer cell invasiveness.^53^ USP7 is a particular deubiquitinating enzyme for ABCB1, which exerts crucial influences over treatment resistance in triple‐negative breast cancer (TNBC). USP7 interacted with ABCB1 directly and controlled its stability.^54^ In our study, interestingly, we found USP7 to be a binding partner for both AKT and USP27X‐AS1 in the current investigation. Furthermore, we observed USP27X‐AS1 increased USP7‐mediated deubiquitylation of AKT and a rise in AKT protein level for the first time.

To elucidate the rationale behind the elevated expression of USP27X‐AS1 in HCC, we employed bioinformatics databases to identify potential proto‐oncogenic transcription factors. Our focus narrowed to the SP1 transcription factor, which is overexpressed in various cancers, associated with poor prognosis, and constitutes a classical downstream feature in the AKT signalling pathway.^55^, ^56^ In this study, we confirmed that SP1 binds to the USP27X‐AS1 promoter, activating USP27X‐AS1 transcription and resulting in elevated expression in HCC tissues. Notably, lncRNAs typically exert their biological functions through physical interactions with DNA, RNA or proteins.^57^ Our investigation revealed that USP27X‐AS1 exacerbates HCC progression by forming a complex with USP7 and AKT, facilitating interaction among the three and diminishing AKT ubiquitination. Ultimately, we validated that the heightened expression of USP27X‐AS1 in HCC stems from the activation of its transcription by the SP1 transcription factor. These findings provide robust supporting evidence and enhance the comprehension of the genetic basis and aetiology of HCC.

## 5 CONCLUSION

**FIGURE 8:**
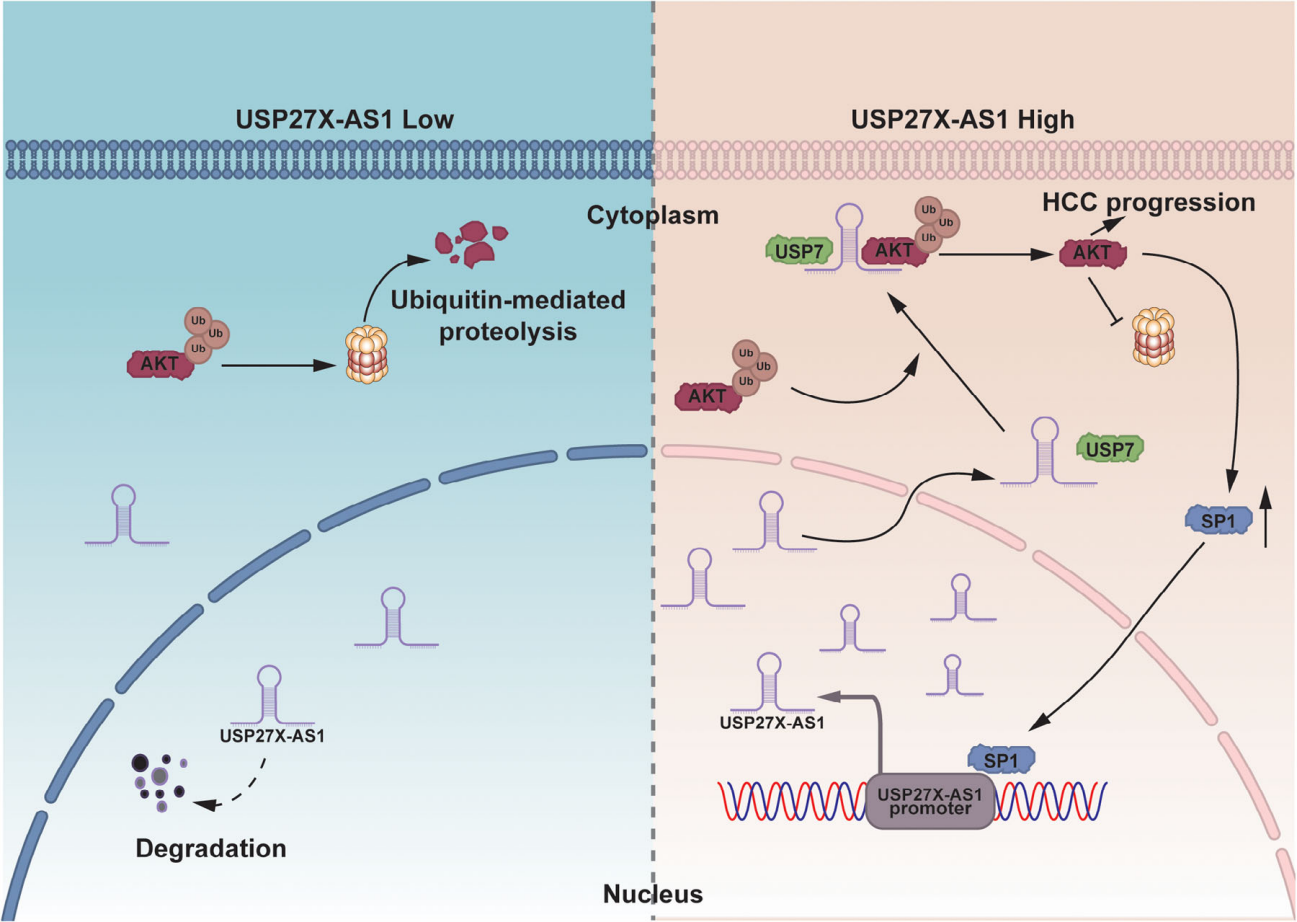
Integrated model diagram depicting how USP27X‐AS1 promotes AKT stabilisation via intensifying interactions between AKT and its novel deubiquitinase USP7 in hepatocellular carcinoma (HCC).

We confirmed that USP27X‐AS1 acts as a novel oncogenic lncRNA in HCC. Mechanistically, USP27X‐AS1 enhances USP7‐mediated deubiquitination and upgrading of AKT to promote HCC progression; we also showed that SP1 binds to the promoter of USP27X‐AS1, enhances USP27X‐AS1 transcription, revealing a novel regulatory axis of SP1‐USP27X‐AS1‐USP7‐AKT in HCC. These findings shed light on HCC treatment and point to USP27X‐AS1 as a potential predictive biomarker and treatment target for the malignancy (Figure 8).

## AUTHOR CONTRIBUTIONS

Chen Su: conceptualised and wrote the manuscript. Jie Mo: participated in manuscript writing and managed the figures. Haoquan Zhang: performed bioinformatics analysis and completed part of the experiments. Zhibin Liao, Bixiang Zhang and Peng Zhu: conceived the study and provided advice. All authors participated in manuscript editing, and read and approved the final version.

## CONFLICT OF INTEREST STATEMENT

All authors declare that there is no conflict of interest related to this manuscript.

## ETHICS STATEMENT

Not applicable.

## FUNDING INFORMATION

National Natural Science Foundation of China, Grant Numbers: 82103597, 81874189 and 81001305.

## Supporting information

Supporting Information

## Data Availability

The data used in the article can be made available by the corresponding author (Peng Zhu) upon reasonable request.
