# Supplementary material for: SP1‐activated USP27X‐AS1 promotes hepatocellular carcinoma progression via USP7‐mediated AKT stabilisation
Source: Clin Transl Med. 2024 Jan 27;14(1):e1563. doi: 10.1002/ctm2.1563 (PMC10819096; doi:10.1002/ctm2.1563)
Supplement: Supplementary file 1 — Supporting Information [file CTM2-14-e1563-s001.docx]

**Supplementary Figures, Figure Legends and Tables**


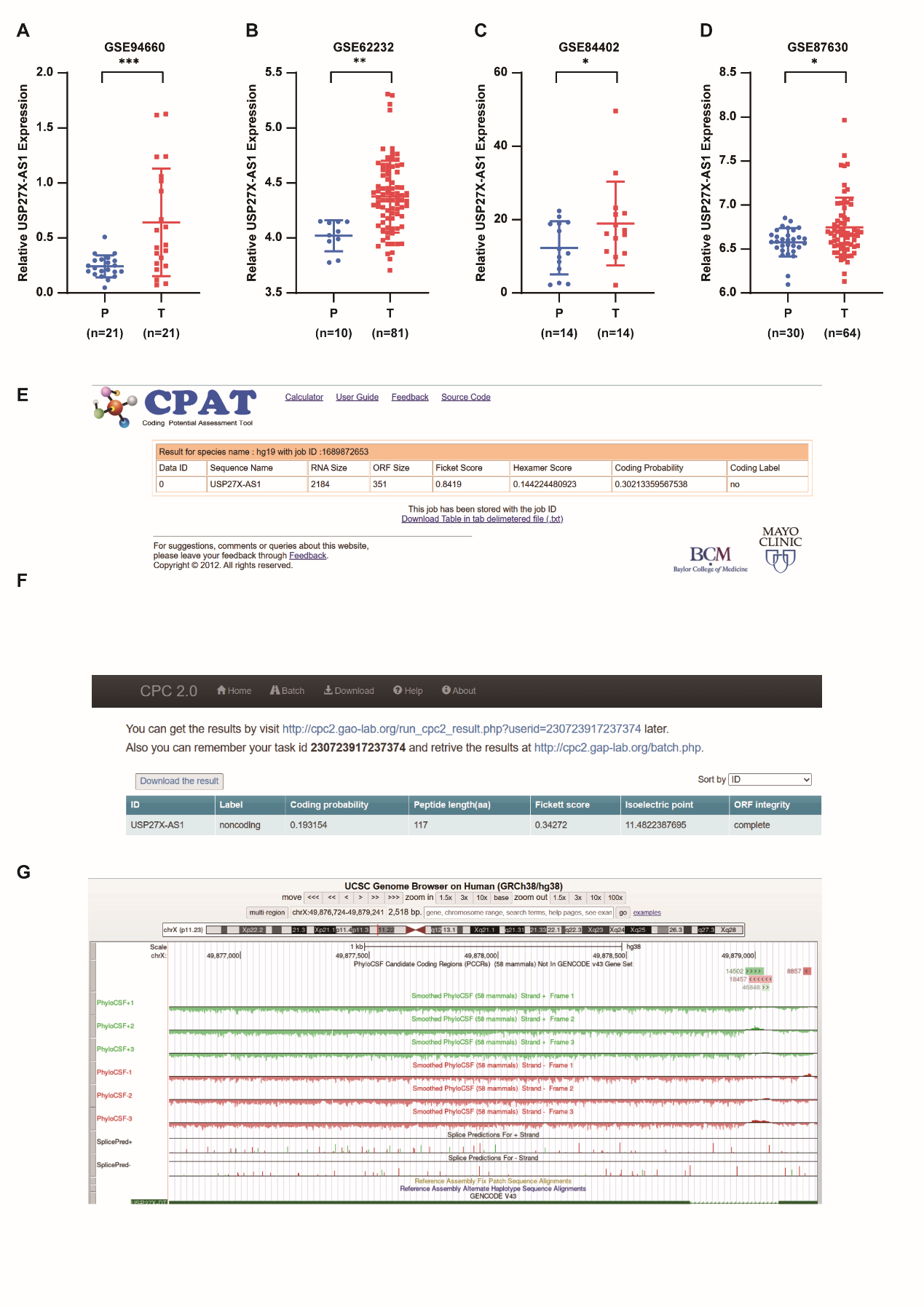


**Figure S1 (Related to Figure 1)**

A-D. The expression of USP27X-AS1 in HCC cohort GSE94660 (A), GSE62232 (B), GSE84402 (C), GSE87630 (D). E-F. Coding-Potential Assessment Tool (E) and The Open Reading Frame Finder online tool (F) prediction for the protein-coding potential of USP27X-AS1. G. UCSC Genome browser depictions of USP27X-AS1 and its conserved analogs.

Data and error bars were shown as mean ± SD of triplicate independent replicate experiments. **P* <0.05, ***P*<0.01, ****P*<0.001, ns: no significance. Data were analyzed by paired Student’s *t* test (A-D).


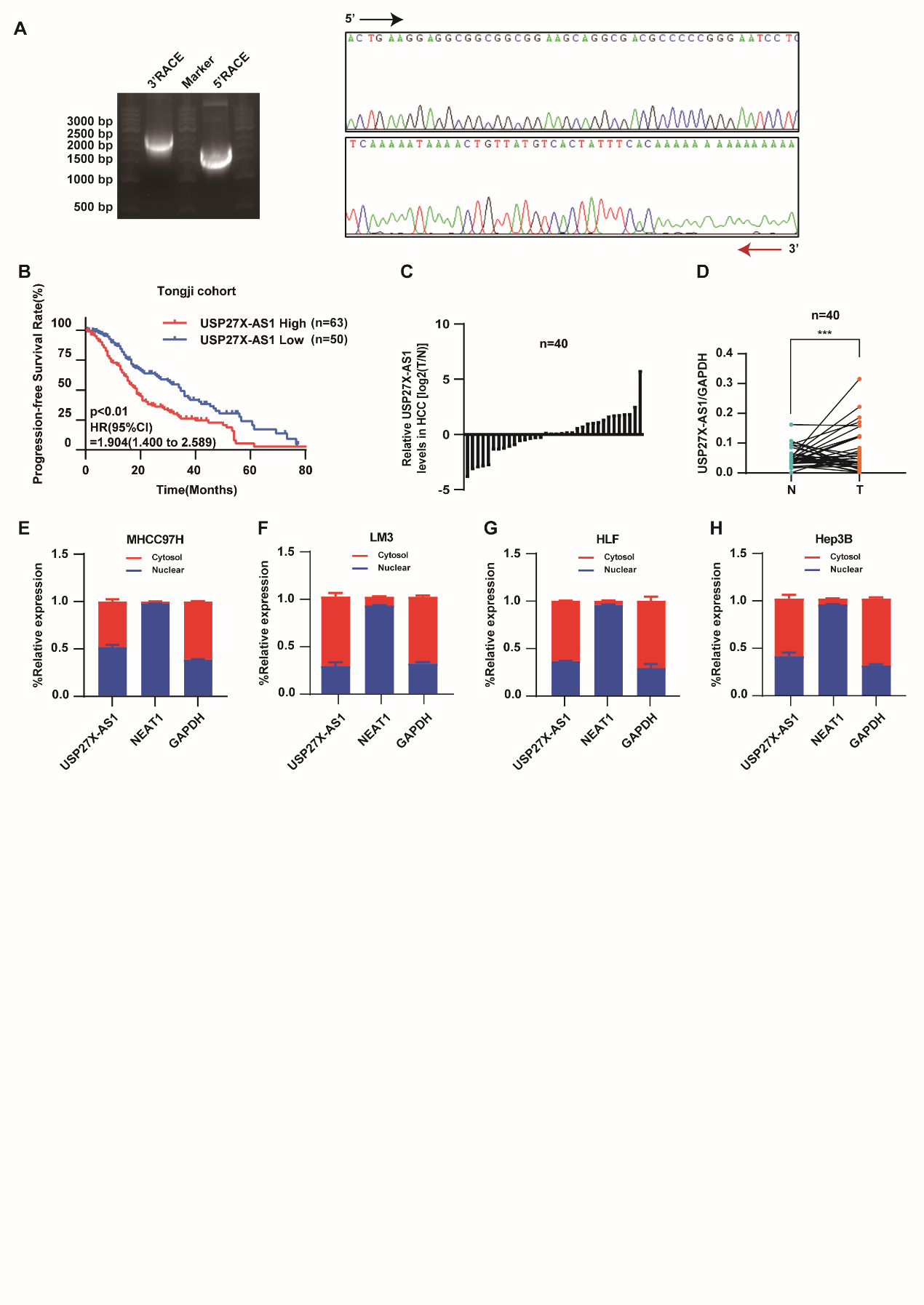


**Figure S2 (Related to Figure 1)**

A. 5’-3’ RACE assay was used to identify the full-length of USP27X-AS1. B. Kaplan-Meier survival analyses of Progression-free Survival (PFS) rate based on USP27X-AS1 expression from Tongji cohort. C-D. The RNA expression level of USP27X-AS1 from 40 paired HCC samples and adjacent non-tumor tissues from Tongji cohort. E-H. The RNA level of USP27X-AS1 in the nucleus and cytoplasm of MHCC97H (E), LM3 (F), HLF (G) and Hep3B (H).

Data and error bars were shown as mean ± SD of triplicate independent replicate experiments. **P* <0.05, ***P*<0.01, ****P*<0.001, ns: no significance. Data were analyzed by paired Student’s *t* test (D). Log-rank test was used for survival comparison (B)


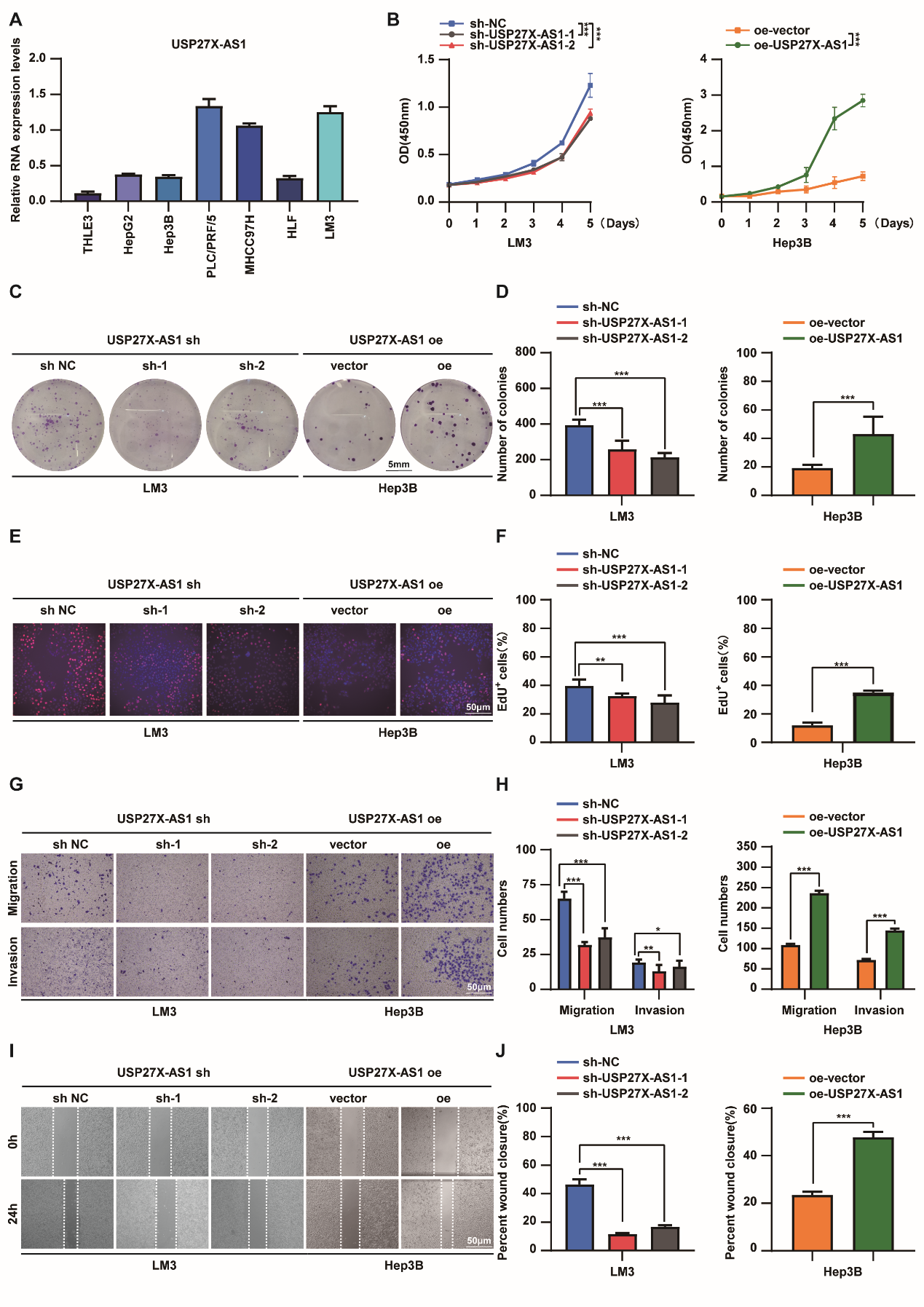


**Figure S3 (Related to Figure 2)**

A. The RNA level of USP27X-AS1 in HCC cell lines. B. The OD (450 nm) value of CCK-8 assay in LM3 knockdown cell line and Hep3B overexpression cell line. C-D. Representative images (C) and number (D) of colony formation assay in LM3 knockdown cell line and Hep3B overexpression cell line. E-F. Representative images (E) and positive cell number (F) of EdU assay in LM3 knockdown cell line and Hep3B overexpression cell line. G-H. Representative images (G) and number (H) of migration or invasion cells in LM3 knockdown cell line and Hep3B overexpression cell line. I-J. Representative images (I) and percentage wound closure (J) of wound healing assay in LM3 knockdown cell line and Hep3B overexpression cell line.

Data and error bars were shown as mean ± SD of triplicate independent replicate experiments. **P* <0.05, ***P*<0.01, ****P*<0.001, ns: no significance. Data were analyzed by paired Student’s *t* test (B, D, F, H and J).


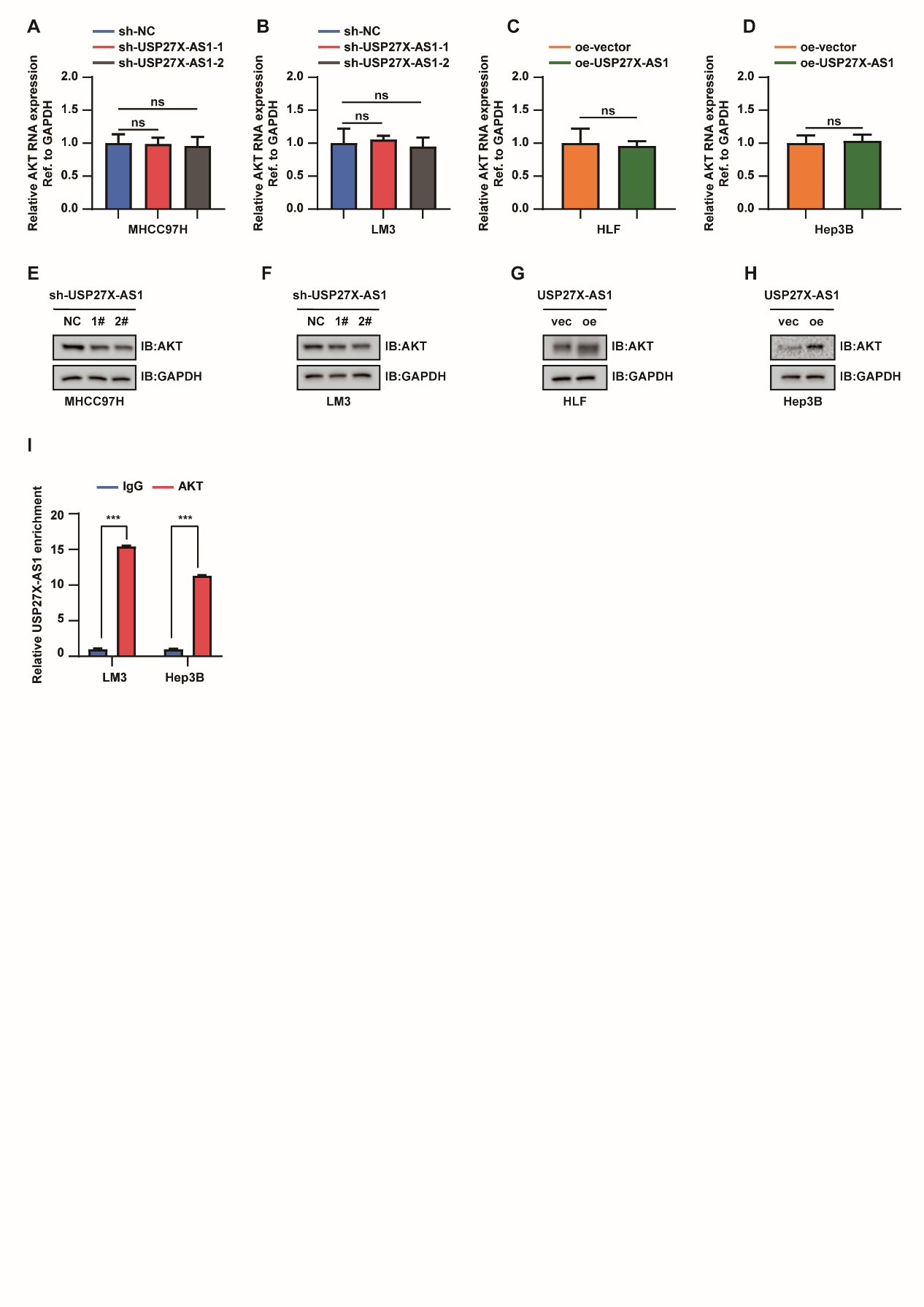


**Figure S4 (Related to Figure 4)**

A-D. The RNA expression of AKT in USP27X-AS1 knockdown or overexpression cells. E-H. Changes of AKT protein expression in USP27X-AS1 knockdown or overexpression cells. I. RIP assay tested the interaction between USP27X-AS1 and AKT in LM3 and Hep3B cells.

Data and error bars were shown as mean ± SD of triplicate independent replicate experiments. **P* <0.05, ***P*<0.01, ****P*<0.001, ns: no significance. Data were analyzed by paired Student’s *t* test (A-D and I).


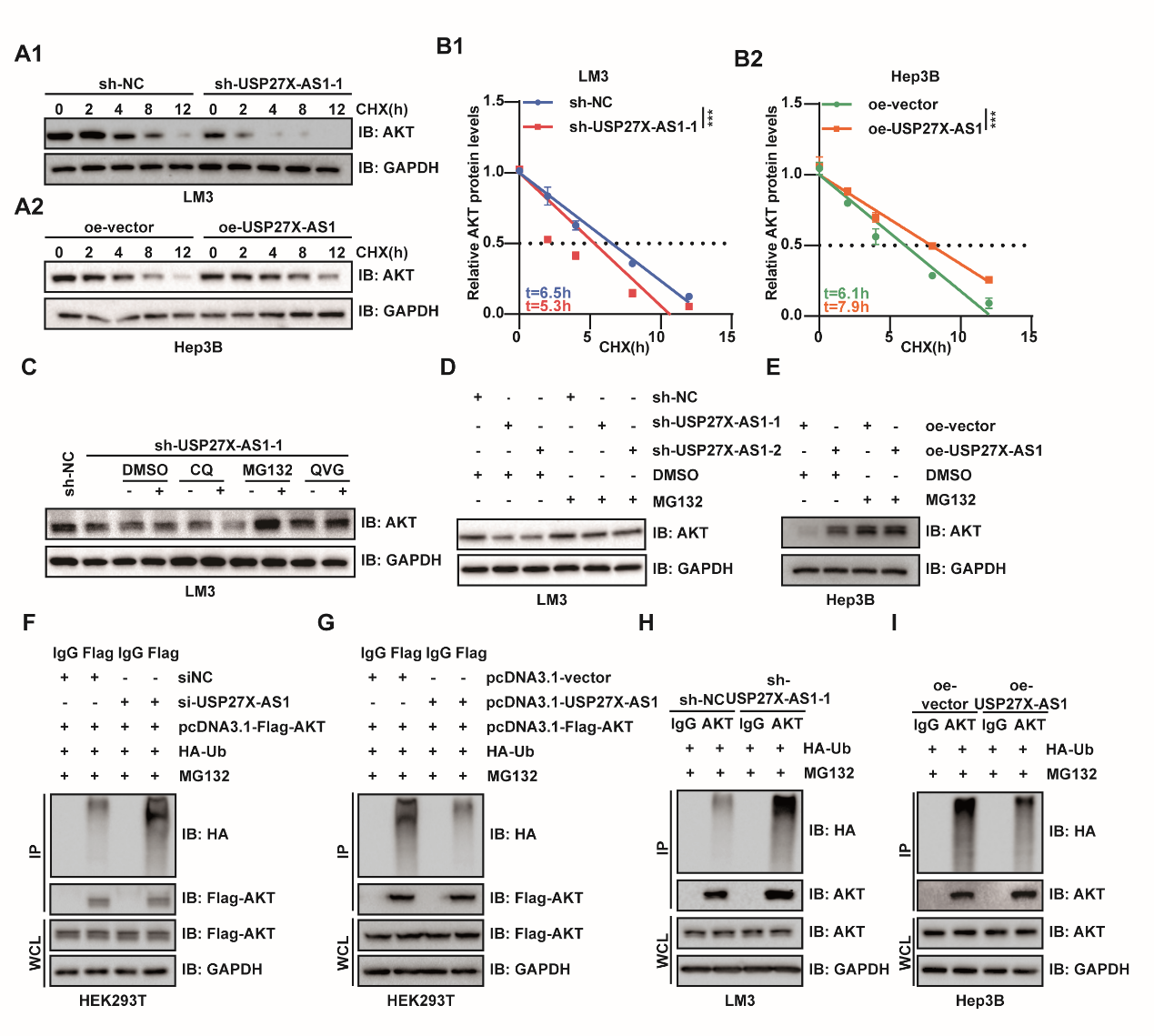


**Figure S5 (Related to Figure 5)**

A-B. Half-life assay tested the change of AKT stability under USP27X-AS1 knockdown (LM3) or overexpression (Hep3B) conditions. C. Changes of AKT protein expression after DMSO (2 μL), CQ (10 μM), MG132 (10 μM) or QVG (10 μM) 6 hours treatment in LM3 USP27X-AS1 knockdown cells. D-E. Changes of AKT protein expression in LM3 USP27X-AS1 knockdown or Hep3B USP27X-AS1 overexpression cells after treating 6 hours with MG132 (10 μM). F-G. Ubiquitination-based exogenous Co-IP detected the changes of AKT total poly-ubiquitination level upon USP27X-AS1 interfered down or overexpression. H-I. Ubiquitination-based endogenous Co-IP detected the changes of AKT total poly-ubiquitination level upon USP27X-AS1 knockdown (LM3) or overexpression (Hep3B).

Data and error bars were shown as mean ± SD of triplicate independent replicate experiments. **P* <0.05, ***P*<0.01, ****P*<0.001, ns: no significance. Data were analyzed by paired Student’s *t* test (B).


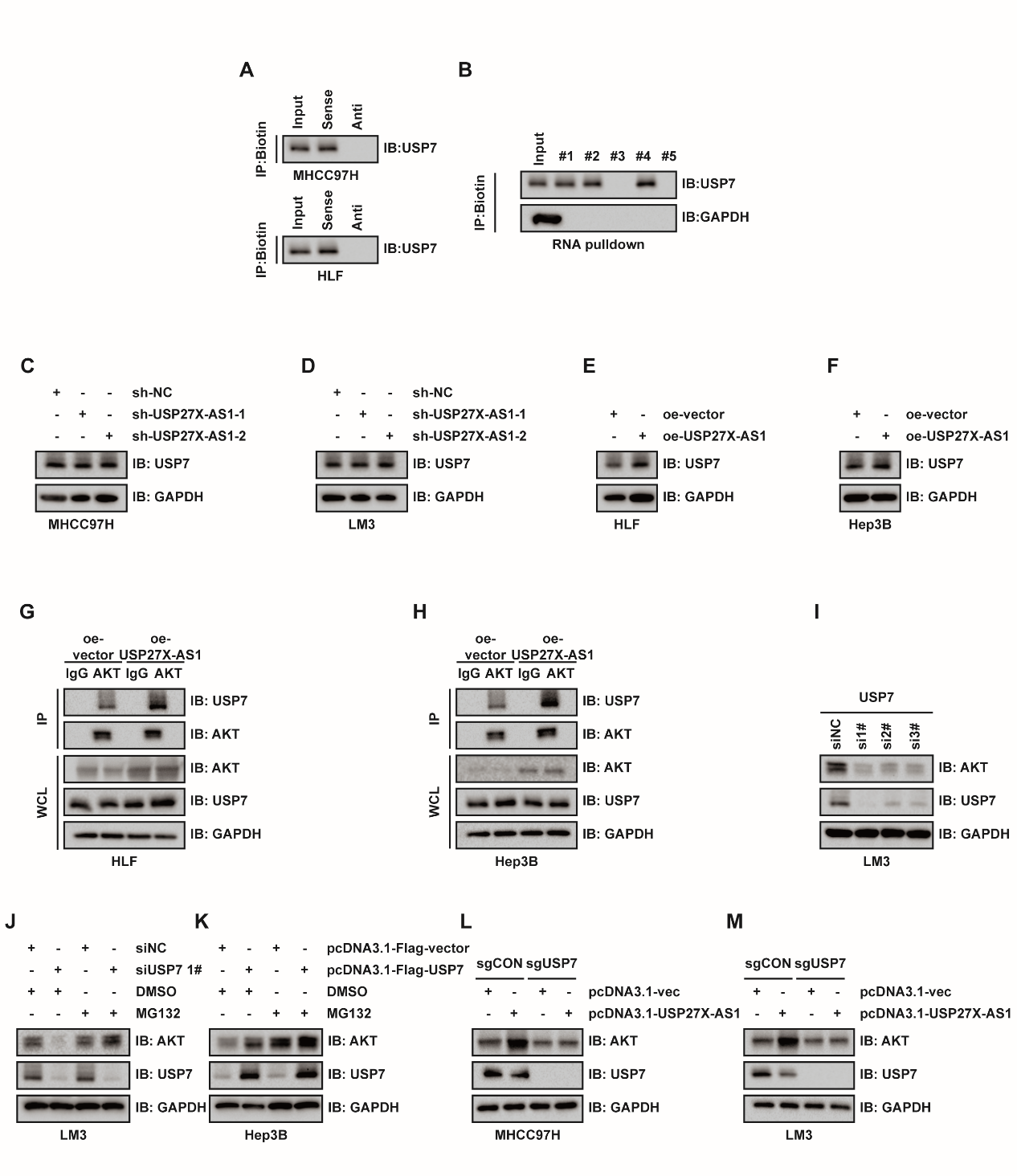


**Figure S6 (Related to Figure 5)**

A. RNA pulldown assay tested the interaction between USP27X-AS1 and USP7 in MHCC97H and HLF cells. B. RNA pulldown assay detected the region of USP27X-AS1 to bind USP7. C-F. Changes of USP7 upon USP27X-AS1 knockdown (MHCC97H, LM3) or overexpression (HLF, Hep3B). G-H. Endogenous Co-IP tested the interaction between USP7 and AKT upon USP27X-AS1 overexpression (HLF, Hep3B). I. Changes of AKT protein expression upon USP7 was interfered in LM3 cells. J-K. Changes of AKT protein expression in MHCC97H interfered with USP7 or HLF USP7 overexpressed cells after treating 6 hours with MG132(10 μM). L-M. Western blot analysis examined the expression of AKT upon USP27X-AS1 overexpression in USP7 knockout cells (MHCC97H, LM3).


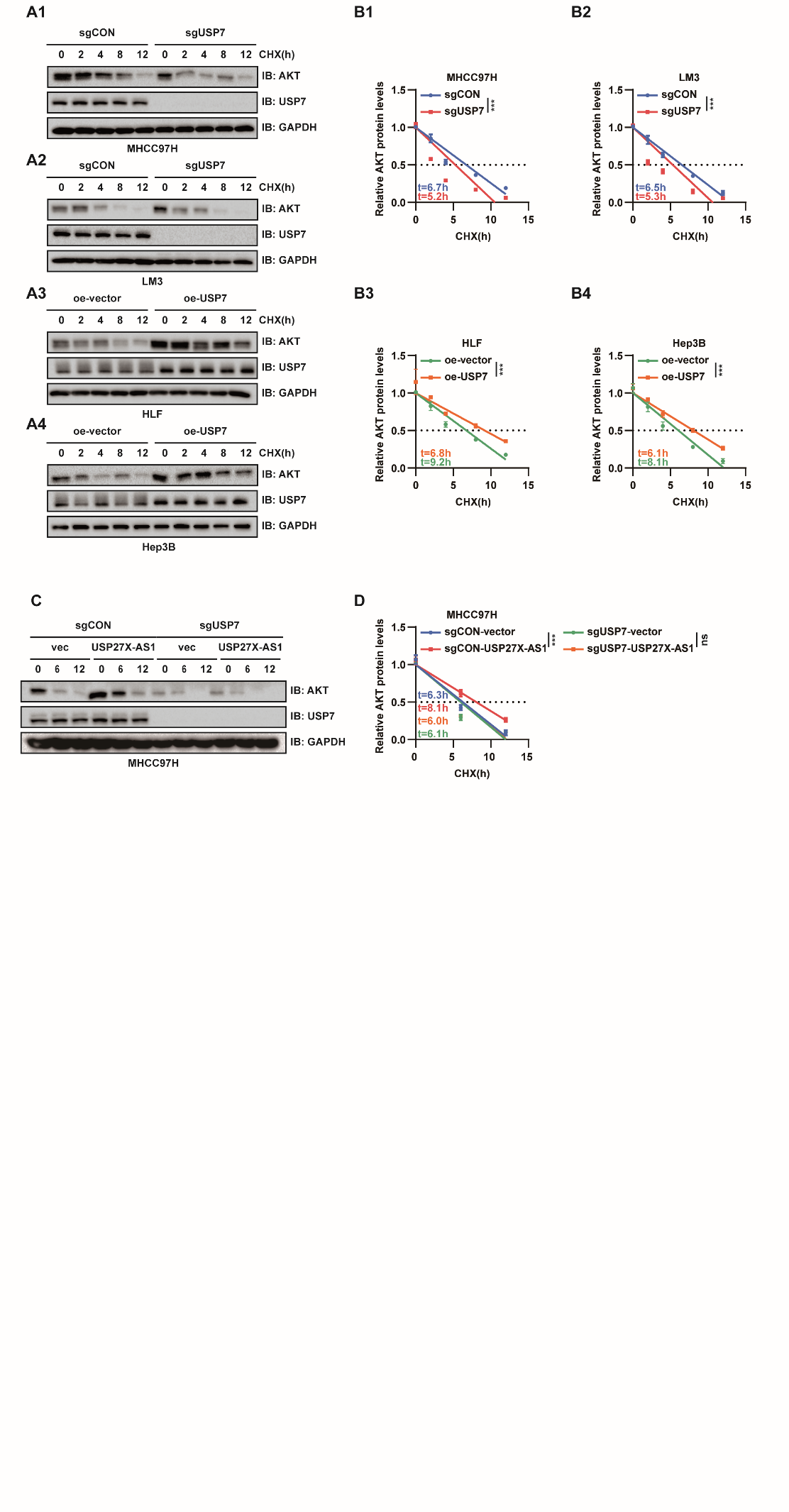


**Figure S7 (Related to Figure 5)**

A-B. Half-life assay tested the stability of AKT upon USP7 knockout (MHCC97H, LM3) or overexpression (HLF, Hep3B). C-D. Half-life assay tested the change of AKT stability in MHCC97H USP27X-AS1 overexpression cells upon USP7 deficiency.

Data and error bars were shown as mean ± SD of triplicate independent replicate experiments. **P* <0.05, ***P*<0.01, ****P*<0.001, ns: no significance. Data were analyzed by paired Student’s *t* test (B and D).


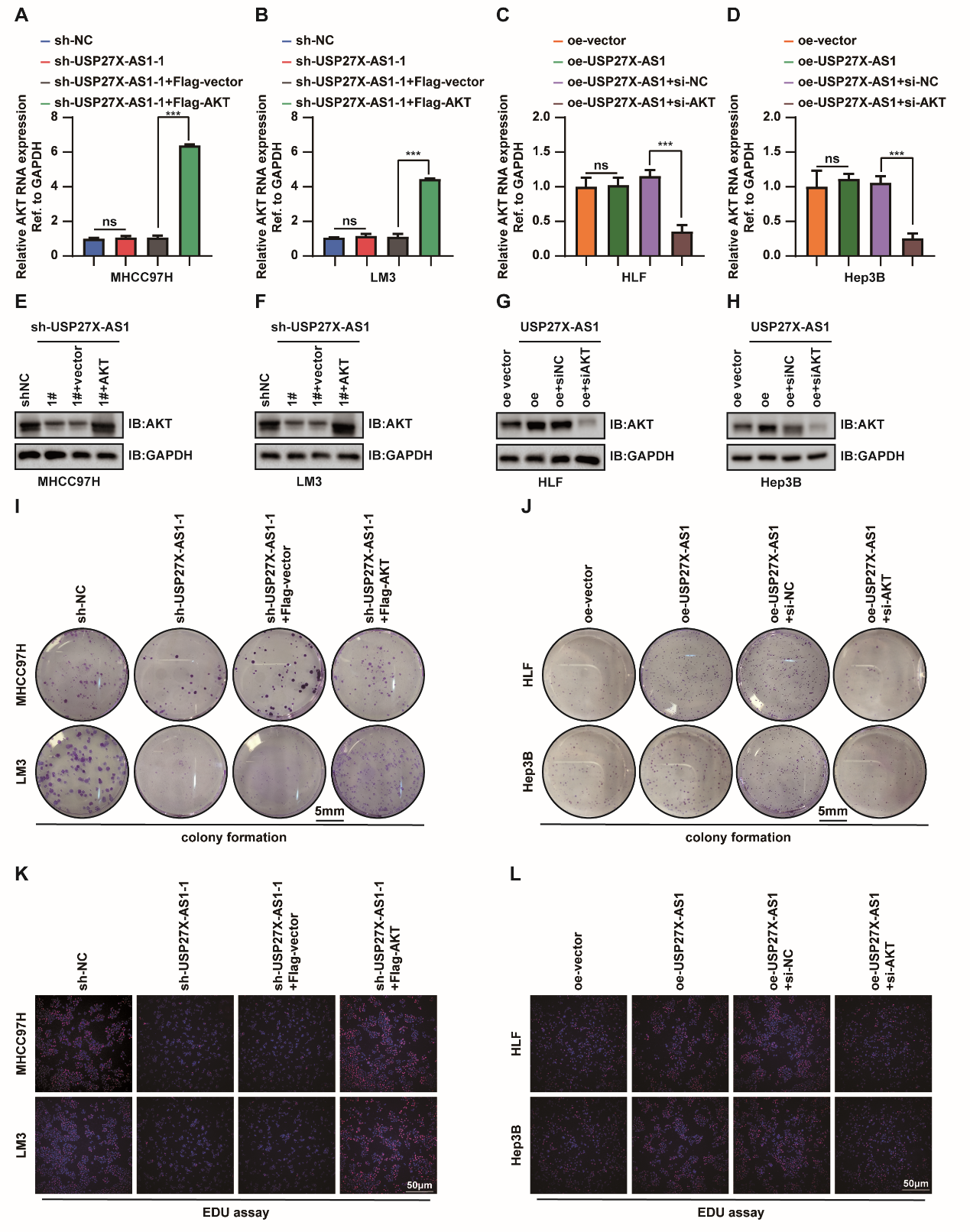


**Figure S8 (Related to Figure 6)**

A-H. qRT-PCR and western blot were used to verify the successful construction of the above-mentioned cell lines. I-J. Representative images of colony formation assay. K-L. Representative images of EdU assay.

Data and error bars were shown as mean ± SD of triplicate independent replicate experiments. **P* <0.05, ***P*<0.01, ****P*<0.001, ns: no significance. Data were analyzed by paired Student’s *t* test (A-D).


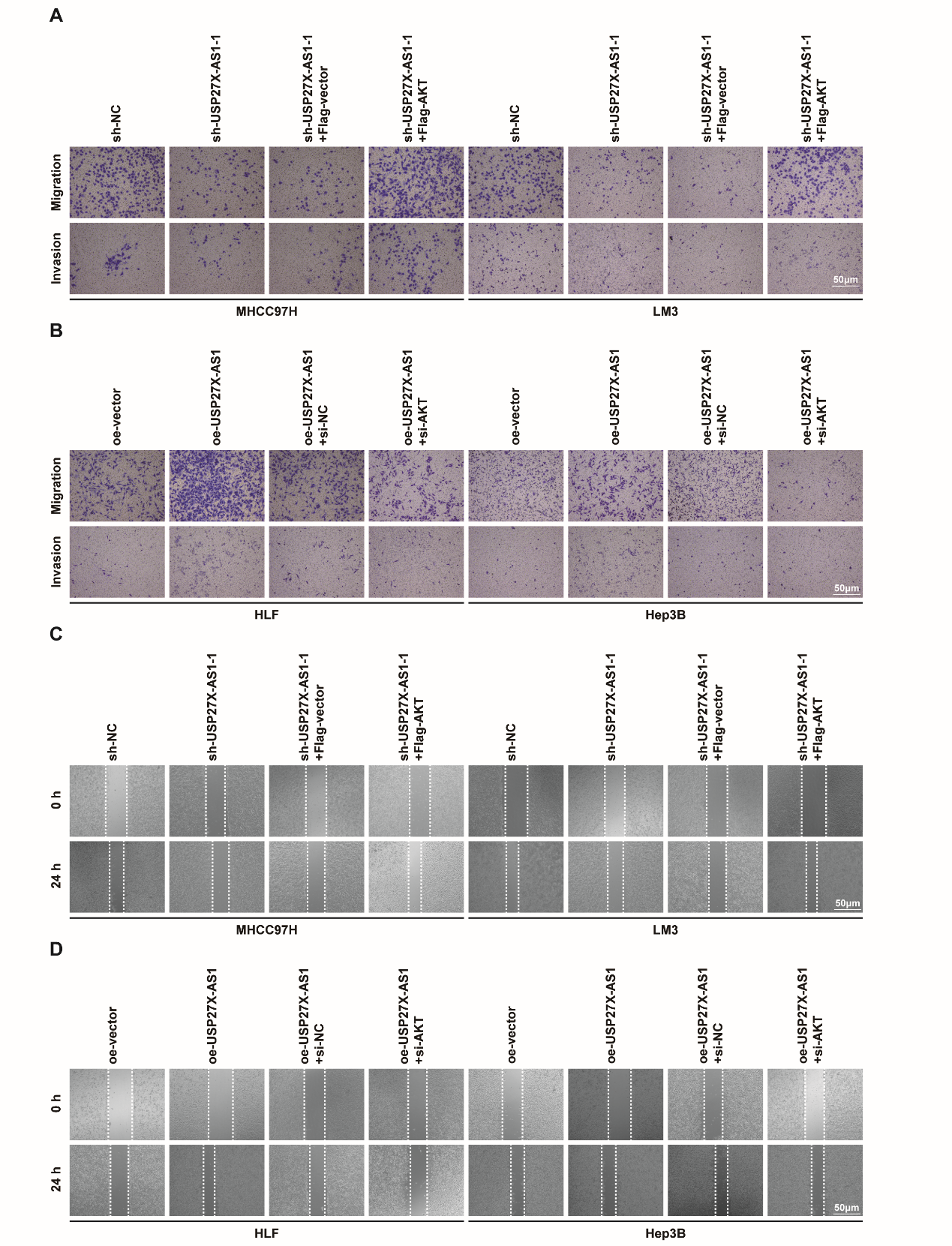


**Figure S9 (Related to Figure 6)**

A-B. Representative images of the migration and invasion cells. C-D. Representative images of wound healing assay.


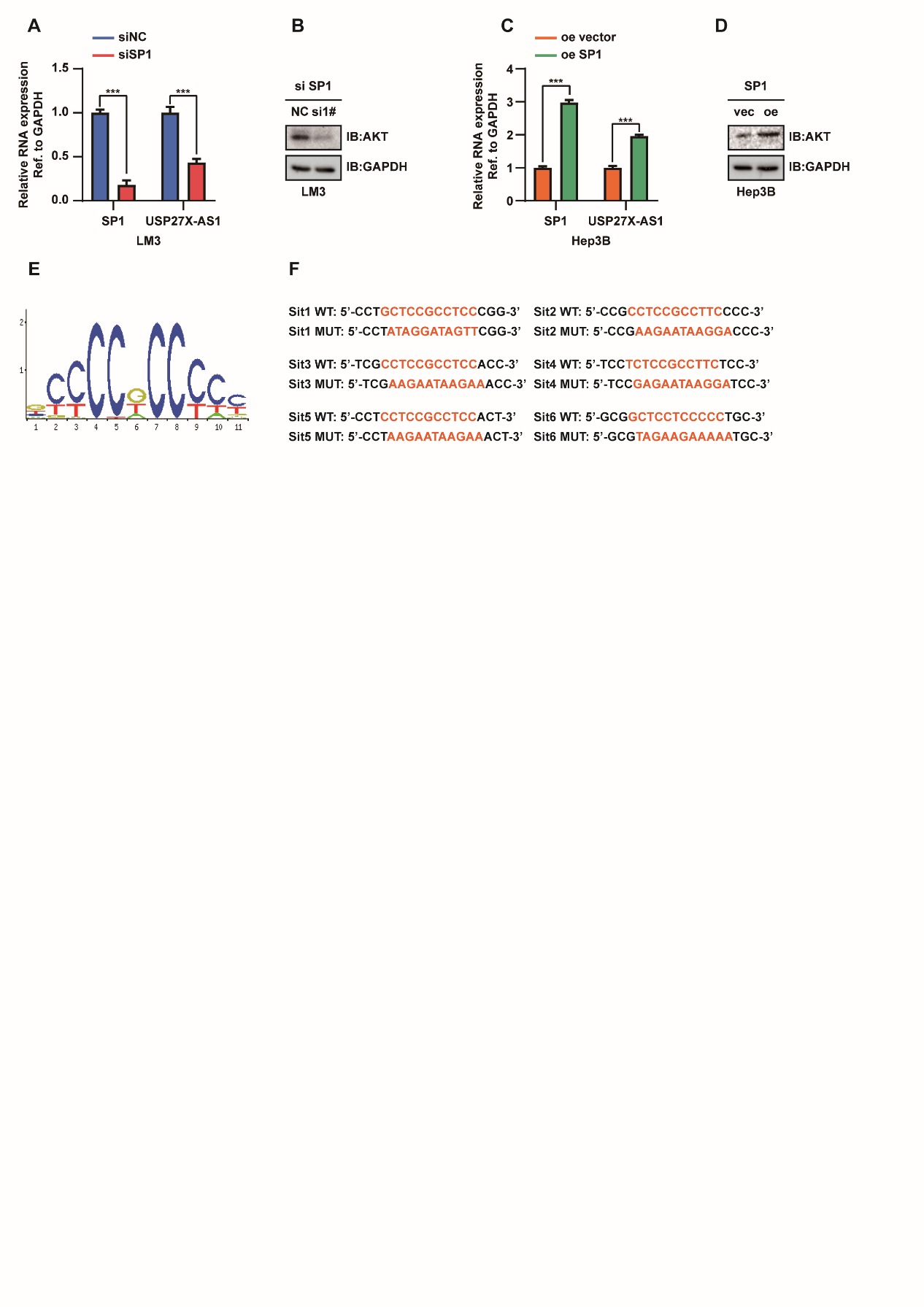


**Figure S10 (Related to Figure 7)**

A. Changes of SP1 and USP27X-AS1 RNA level after SP1 expression was interfered in LM3. B. Changes of AKT protein level after SP1 expression was interfered in LM3. G. Changes of SP1 and USP27X-AS1 RNA level after SP1 overexpression in Hep3B. H. Changes of AKT protein level after SP1 overexpression in Hep3B. E-F. Putative SP1-binding site within the genomic sequence adjacent to e transcription start site of USP27X-AS1 gene.

Data and error bars were shown as mean ± SD of triplicate independent replicate experiments. **P* <0.05, ***P<*0.01, ****P*<0.001, ns: no significance. Data were analyzed by paired Student’s *t* test (A and C).

**Table-S1: qRT-PCR Primer**

| **Primer** | **sequence** |
| --- | --- |
| USP27X-AS1-F | CGATGGCACAATCCACCTCT |
| USP27X-AS1-R | CAGGGCTTAAGGGTCTTGGG |
| NEAT1-F | GGGACAACATTGACCAACGC |
| NEAT1-R | GAAGAAGGGGTGGAGTGAGC |
| AKT-F | AGCGACGTGGCTATTGTGAAG |
| AKT-R | GCCATCATTCTTGAGGAGGAAGT |
| GAPDH-F | CTGGGCTACACTGAGCACC |
| GAPDH-R | AAGTGGTCGTTGAGGGCAATG |
| SP1-F | TGGCAGCAGTACCAATGGC |
| SP1-R | CCAGGTAGTCCTGTCAGAACTT |
| RIP-USP27X-AS1-F | CCTGCAGAAGCCCATAGGTC |
| RIP-USP27X-AS1-F | CTTGGTACATCTGGCCCTCG |
| CUT&RUN-USP27X-AS1-FL-F | GTGAATGTGTTTCTCCGT |
| CUT&RUN-USP27X-AS1-FL-R | TCAGAATCTCGAAGGGAGGT |
| CUT&RUN-USP27X-AS1-sit1-F | AACCTCGTCGCTGT |
| CUT&RUN-USP27X-AS1-sit1-R | CGGGTTCGGGCTCCAGC |
| CUT&RUN-USP27X-AS1-sit2-F | GTGGCGTCCACCTTCCC |
| CUT&RUN-USP27X-AS1-sit2-R | CTTCCCCTCCACCTTCG |
| CUT&RUN-USP27X-AS1-sit3-F | CTCCGCCTTCCCCTCC |
| CUT&RUN-USP27X-AS1-sit3-R | CGCCGTCTCCGCCTCCTC |
| CUT&RUN-USP27X-AS1-sit4-F | GCCTTCTCCGCCGTCTC |
| CUT&RUN-USP27X-AS1-sit4-R | ACTGGCGGCGGCTCCTCCC |
| CUT&RUN-USP27X-AS1-sit5-F | TACCTCCTCCGCCTCCACTG |
| CUT&RUN-USP27X-AS1-sit5-R | CACTGAAGGAGGCGGC |
| CUT&RUN-USP27X-AS1-sit6-F | ACTTTCACTGAAGGAGGCGG |
| CUT&RUN-USP27X-AS1-sit6-R | TTAAAGGAAACGGGGCCTGG |

**Table-S2: Related sequence information for plasmid construction**

| **Primer** | **sequence** |
| --- | --- |
| sh-USP27X-AS1-1-F | CCGGGCTGTGCTGCCTGAGTATACTCGAG TATACTCAGGCAGCACAGC TTTTTG |
| sh-USP27X-AS1-1-R | CAAAAAGCTGTGCTGCCTGAGTATACTCG  AGTATACTCAGGCAGCACAGCCCGG |
| sh-USP27X-AS1-2-F | CCGGGCTCTTGCTTCAATTCCTTCTCGAG AAGGAATTGAAGCAAGAGC TTTTTG |
| sh-USP27X-AS1-2-R | CAAAAAGCTCTTGCTTCAATTCCTTC  TCGAGAAGGAATTGAAGCAAGAGCCCGG |
| oe-USP27X-AS1-F | Cttggtaccgagctcggatcc  ACTGAAGGAGGCGGCGGC |
| oe-USP27X-AS1-R | Tgctggatatctgcagaattc  TTTTTTTTTTTTTTTGTGAAATAGTGAC |
| oe-AKT-F | Cttggtaccgagctcggatcc  ATGAGCGACGTGGCTATTGTG |
| oe-AKT-R | Tgctggatatctgcagaattc  TCAGGCCGTGCCGCTGGC |
| oe-USP7-F | Cttggtaccgagctcggatcc  ATGAACCACCAGCAGCAGCA |
| oe-USP7-R | Tgctggatatctgcagaattc  TCAGTTATGGATTTTAATGGCCTTT |
| sgUSP7-F | GCGGAGGAGGACATGGAGGA NGG |
| sgUSP7-R | NGG TCCTCCATGTCCTCCTCCG |
| oe-SP1-F | Cttggtaccgagctcggatcc  ATGAGCGACCAAGATCACTCCA |
| oe-SP1-R | Tgctggatatctgcagaattc  TCAGAAGCCATTGCCACTGAT |
| FISH-USP27X-AS1 | CgggAAgCgCAggACCTgATCA |

**Table-S3: Other primer**

| **Primer** | **Sequence** |
| --- | --- |
| si-USP27X-AS1-F | GCTGTGCTGCCTGAGTATA |
| si-USP27X-AS1-R | TATACTCAGGCAGCACAGC |
| si-USP7-1-F | GAATGACATGTACGATGAA |
| si-USP7-1-R | TTCATCGTACATGTCATTC |
| si-USP7-2-F | GAGCGACCTTACCCAAGTT |
| si-USP7-2-R | AACTTGGGTAAGGTCGCTC |
| si-USP7-3-F | TAAGGACCCTGCAAATTAT |
| si-USP7-3-R | ATAATTTGCAGGGTCCTTA |
| si-AKT-F | CTCACCCAGTGACAACTCA |
| si-AKT-R | TGAGTTGTCACTGGGTGAG |
| si-SP1-F | GCAACTCATTGCTGCTATTT |
| si-SP1-R | ATAGCAGCAATGAGTTGCTT |
| Negative control-sense | TTCTCCGAACGTGTCACGTTT |
| Negative control-antisense | ACGTGACACGTTCGGAGAATT |

**Table-S4 Antibody infomation**

| **Antibody** | **Company** | **Cat No.** | **Concentration** |
| --- | --- | --- | --- |
| Flag-tag Mouse mAb | sigma | MA1-91878 | 1:2000 (For WB/IP) |
| HA-Tag (C29F4) Rabbit mAb | Cell Signaling Technology | #3724 | 1:2000 (For WB/IP) |
| Myc-Tag (9B11) Mouse mAb | Cell Signaling Technology | #2276 | 1:1000 (For WB/IP) |
| GAPDH Antibody | ABclonal | AC033 | 1:50000 (For WB) |
| AKT (C73H10) Rabbit mAb | Cell Signaling Technology | #2938 | 1:1000 (For WB/IP) |
| PCNA (D3H8P) XP® Rabbit mAb | Cell Signaling Technology | #13110 | 1:200 (For IHC) |
| Anti-biotin, HRP-linked Antibody | Cell Signaling Technology | #7075 | 1:500 (For RNA pulldown) |
| HAUSP (D17C6) XP® Rabbit mAb | Cell Signaling Technology | #4833 | 1:1000 (For WB/IP) |

**Table-S5: Correlation between the USP27X-AS1 and clinicopathologic characteristics in patients from HCC**

| **Clinicopathological**  **variables** | **Relative USP27X-AS1 Expression** | | **P value** |
| --- | --- | --- | --- |
|  | **Low (55)** | **High (58)** |  |
| **Gender**  Male  Female | 46  9 | 47  11 | 0.344 |
| **Age**  ≤50  > 50 | 34  21 | 32  26 | 0.500 |
| **AFP (ug/L)**  ≤400  > 400 | 32  23 | 24  34 | **0.033** |
| **ALT(ng/ml)**  ≤75  >75 | 48  7 | 54  4 | 0.656 |
| **Cirrhosis**  No  Yes | 20  35 | 17  41 | 0.795 |
| **Tumor size (cm)**  ≤5  >5 | 25  30 | 14  44 | **0.016** |
| **Tumor number**  Single  Multiple | 41  14 | 42  16 | 0.489 |
| **Vascular invasion**  No  Yes | 49  6 | 38  20 | **0.013** |
| **BCLC stage**  0+A  B+C | 41  14 | 26  32 | **0.004** |
|  |  |  |  |

**Table-S6 USP27X-AS1univariate and multivariate analyses of factors associated with OS for patients from HCC**

| **Clinicopathological**  **variables** | **Univariable** | | **Multivariable** | |
| --- | --- | --- | --- | --- |
|  | **HR (95% CI)** | ***P* value** | **HR (95% CI)** | ***P* value** |
| **Age (>=60/<60)** | 2.791  （0.272,2.879） | 0.549 |  |  |
| **Gender (Male/Female)** | 0.651  （0.325,1.302） | 0.244 |  |  |
| **Size(>=5cm/<5cm)** | 2.532（0.396,3.948） | **0.023** | 5.0683  (2.667,7.685) | 0.062 |
| **Tumor differentiation** | 3.423（1.239,4.738） | **0.047** |  |  |
| **Vein-invasion (Yes/No)** | 1.034（0.338,1.989） | 0.942 |  |  |
| **TNM (III -IV/ I- II)** | 4.429（2.682,7.312） | **0.038** |  |  |
| **Lymph-node metastasis (Yes/No)** | 4.751（2.838,7.955） | **＜0.001** | 2.757  (1.574,4.913) | **0.001** |
| **USP27X-AS1**  **expression** | 7.367  (1.296,12.139) | **0.032** | 5.115  (0.358,10.675) | **0.038** |
